# Developing a framework for evaluating the impact of Healthcare Improvement Science Education across Europe: a qualitative study

**DOI:** 10.3352/jeehp.2017.14.28

**Published:** 2017-11-29

**Authors:** Manuel Lillo-Crespo, M. Cristina Sierras-Davó, Rhoda MacRae, Kevin Rooney

**Affiliations:** 1 Nursing Department, Faculty of Health Sciences, University of Alicante, Alicante, Spain; 2 Institute for Healthcare Policy and Practice, School of Health Nursing and Midwifery, The University of the West of Scotland, Hamilton, UK; 3 Department of Anaesthesia and Intensive Care Medicine, Royal Alexandra Hospital, Paisley, UK; Hallym University, Korea

**Keywords:** Europe, Health personnel, Curriculum, Delivery of health care, Students

## Abstract

**Purpose:**

Frontline healthcare professionals are well positioned to improve the systems in which they work. Educational curricula, however, have not always equipped healthcare professionals with the skills or knowledge to implement and evaluate improvements. It is important to have a robust and standardized framework in order to evaluate the impact of such education in terms of improvement, both within and across European countries. The results of such evaluations will enhance the further development and delivery of healthcare improvement science (HIS) education. We aimed to describe the development and piloting of a framework for prospectively evaluating the impact of HIS education and learning.

**Methods:**

The evaluation framework was designed collaboratively and piloted in 7 European countries following a qualitative methodology. The present study used mixed methods to gather data from students and educators. The framework took the Kirkpatrick model of evaluation as a theoretical reference.

**Results:**

The framework was found to be feasible and acceptable for use across differing European higher education contexts according to the pilot study and the participants’ consensus. It can be used effectively to evaluate and develop HIS education across European higher education institutions.

**Conclusion:**

We offer a new evaluation framework to capture the impact of HIS education. The implementation of this tool has the potential to facilitate the continuous development of HIS education.

## Introduction

Healthcare improvement science (HIS) constitutes a body of knowledge, as well as a strategic dimension aligned to the eHealth Action Plan 2012–2020 as a roadmap for achieving smart and sustainable healthcare systems across Europe [1]. The impact and effectiveness of HIS education must therefore be characterized. For this reason, a team led by the Faculty of Health Sciences at the University of Alicante (Spain) as an Improvement Science Training for European Healthcare Workers (ISTEW) European Commission Project partner began developing an evaluation framework designed to be used by higher education institutions [2-4]. This framework is expected to capture the impact of the HIS educational modules delivered by the ISTEW project. The aim of this paper was to outline the process of development, the resultant framework, and its piloting, and our results will enable continuous evaluation within and across all partner countries.

## Methods

**Fig. 1.**
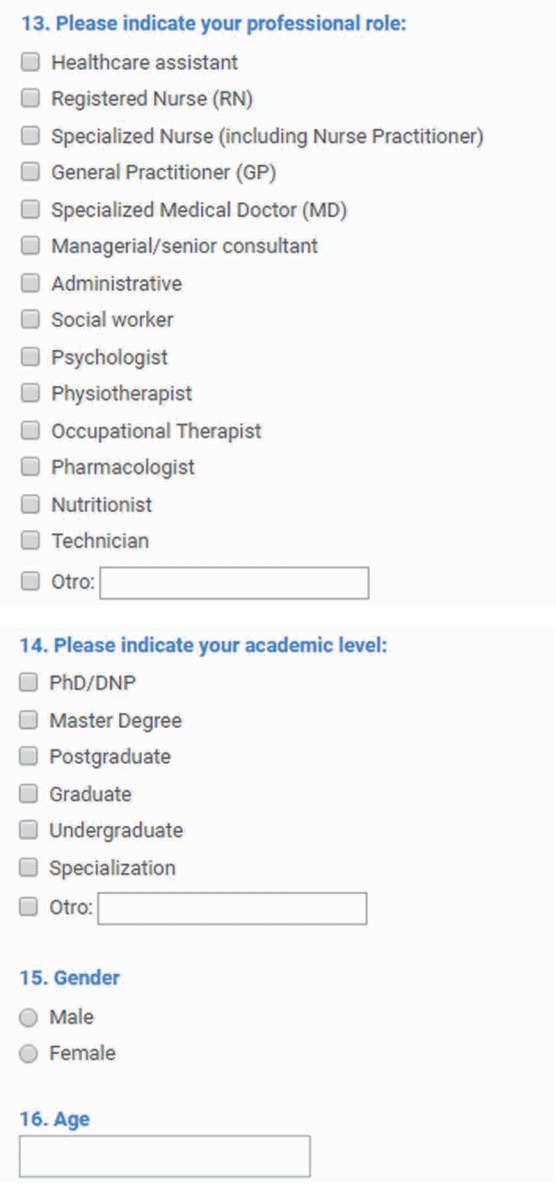
Example of the minimum data set that was developed.

A qualitative mixed-methods methodology was used and divided into 2 steps: the first step corresponded to the development of the framework, and the second step involved its pilot study in different European contexts. The first stage comprised 2 elements: the gathering of a minimum data set (MDS) with the main variables or items corresponding to the educational module selected and a number of questionnaires designed for different participants at each stage of the learning process (Fig. 1) that were unified by the ISTEW teams from different countries and contexts through consensus. Kirkpatrick’s 4-level training evaluation model was the conceptual reference used to develop the specific methodology [5,6]. Once the HIS evaluation framework was agreed upon by the ISTEW partnership network composed of 7 teams, a pilot validation (the second step) using the case study method was undertaken across 7 different European educational contexts (Scotland, England, Romania, Slovenia, Italy, Poland, and Spain). The pilot validation was conducted and coordinated by the Spanish team. The total pilot sample was made up of 10 cases. Each case corresponded to a training program involving HIS in the different contexts, and all the selected programs were HIS-related or contained elements of HIS. Participants within each case were selected by convenience and contacted through face-to-face meetings or email by each partner team. The pilot sample came from the following areas: nursing (n=4), medicine (n=3), and psychology (n=3). Participants’ demographics and HIS background were identified through a short set of questions on the first page of the questionnaire (Fig. 1). This set of questions was developed in the first step using the MDS method (Table 1). All data were collected in a classroom in paper format in the beginning, and later using Google Forms remotely. A short introduction was provided, explaining all research goals and objectives, as well as the relationship of the interviewers to the project.

### Ethical approval

Informed consent was provided by the subjects. This study was approved by the Institutional Review Board of the University of Alicante, Spain (IRB Number: 539194-LLP-1-2013-1).

## Results

Based on consensus, the 7 partner teams made the following decisions for developing the HIS framework (first step) and conducting a pilot content validation (second step).

### Selecting a conceptual framework: the Kirkpatrick model

During the construction of the HIS evaluation framework, the partners agreed to use the 4 levels described in the Kirkpatrick model, although we added a fifth level to evaluate the return on investment. Without level 5, Kirkpatrick’s assumption ignores the potential differences involving training and training outcomes that may exist among key stakeholder groups (e.g., trainees, managers, and trainers) in organizations. Moreover, level 5 helps link the learning intervention with the outcomes in context, in relation with the cost-efficiency potentially achieved due to the HIS training programs undertaken by students.

### Developing a minimum data set

According to some authors [7], the original Kirkpatrick model presents an oversimplified view of training effectiveness, because it does not consider individual or contextual influences on the evaluation of training. Therefore, characteristics of the organization, work environment, and the individual trainee are crucial input factors. To fill this gap, an MDS was developed to capture a set of information with uniform categories concerning a specific dimension [8]. The first page of each questionnaire was designed to capture the characteristics of the organization, environment, and student. It was created to incorporate contextual information, in light of the above limitation of the Kirkpatrick model [9].

### Developing the questionnaires

The HIS evaluation framework was designed to be an anonymous self-completed questionnaire with an informed consent page at the beginning. Each questionnaire had open and closed questions and Likert scales. Different questionnaires were developed to capture each level of the learning process. Each respondent also had to create his or her own code, to be used at all levels. This led to the design of 5 different questionnaires for each key stakeholder group. Overall, the framework was designed to capture the impact of the different stages of the HIS learning process, from level 1 (reaction) to level 5 (return on investment).

The framework prospectively captured the outcomes and impacts of HIS education on learners, educators, and healthcare professionals, such as mentors or managers of the learners, in practice settings. Fig. 1 illustrates how the questionnaires were matched to participants.

### Developing the healthcare improvement science evaluation framework and piloting process

**Fig. 2.**
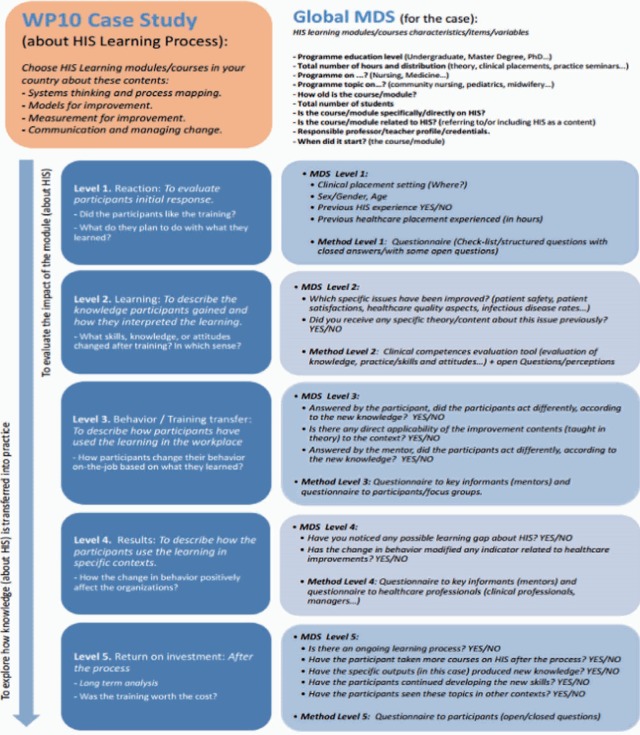
Development of the HIS evaluation framework. HIS, healthcare improvement science; MDS, minimum data set.

**Fig. 3.**
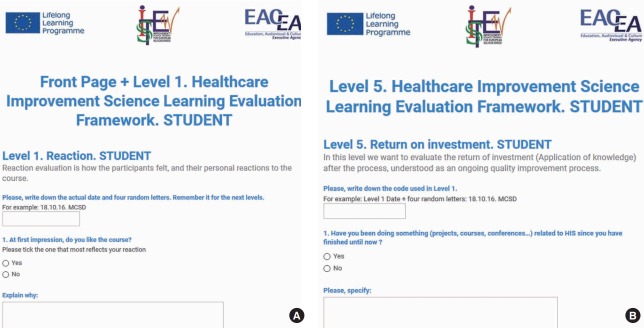
Examples of the online healthcare improvement science learning evaluation framework, level 1 (A) and level 5 (B).

Using the conceptual framework selected as a reference and the methodological process explained above, the ISTEW team arrived at a consensus in terms of the most appropriate levels for evaluating HIS learning, the questionnaires designed and piloted to do so, and what constituted the HIS framework itself. The partner teams completed a pilot content validation of the agreed-upon HIS evaluation framework. The raw data are available in Supplement 1. The piloting process tested the content, understanding, and usability of both the MDS and the various questionnaires. The piloting process was iterative, and successive drafts were produced and refined over time, resulting in a version that was acceptable, feasible, and suitable for use in all 7 countries. After each version, all partners shared ideas for improvement, and students’ comments were taken into account. Some parts of the questionnaires developed as part of the framework construction are shown in Figs. 2 and 3. The piloting process revealed that the more succinct questions and questionnaires were, the easier it was for participants from the 7 different countries and educational contexts to complete them. Over time, the questions became shorter and the questionnaires less complex, with more signposting and explanatory text to promote completion.

## Discussion

The framework composed by the HIS levels and the HIS evaluation framework questionnaires for each level provides a standardized design that overcomes some of the limitations discussed by other authors regarding the design and delivery of interventions through a multicultural European pilot program, taking into account different educational contexts. A previous study suggested using a combination of qualitative and quantitative methods to permit the determination of how context-level factors might modify the effectiveness of an intervention [10]. The methods used in the development of the HIS evaluation framework included participants’ qualitative and quantitative data obtained through the MDS and different questionnaires as well as the discussions and inter-organizational networks among the 7 ISTEW partner teams. A mixed-methods approach analyzing both qualitative and quantitative data, obtained through the MDS and a modified Kirkpatrick evaluation, enabled us to measure the effectiveness of training in various contexts in light of current challenges.

Although there is no doubt that the Kirkpatrick model has made valuable contributions to the theory and practice of evaluating training, the members of the ISTEW partnership were aware of the limitations of such a model, which have implications for the ability of training evaluators to deliver benefits and to further the interests of organizational clients. The limitations highlighted by some authors [9] include “the incompleteness of the model, the assumption of causality, and the assumption of increasing importance of information as the levels of outcomes are ascended.” The ISTEW partnership aimed to adapt and apply the Kirkpatrick model further so that it could overcome those limitations.

Some of the limitations highlighted by other authors associated with the 4 different stages in the Kirkpatrick model [11] were also discussed by the ISTEW partners during the piloting process. Consequently, the partners designed individual questionnaires for different participants to enable them to answer anonymously, with the aim of reducing reticence or concerns about participation. This gave the evaluators the opportunity to provide additional support for learners when they felt that their objectives were not met.

Kirkpatrick and Kirkpatrick [9] questioned how evaluators control for other factors that may affect the impact of the training intervention; in other words, how can we be sure that the module selected is precisely the training intervention needed? Following the recommendation of Øvretveit [12] to try to overcome this by using a tool to measure the ability provided by the modules before and after the event, the HIS evaluation framework included both quantitative and qualitative methods.

An opportunity for future learning would be to test the tool itself on the new HIS modules that were developed due to a delay in the integration of the HIS modules into educational practice. Instead, the pilot sample involved programs or modules already in use that contained elements of HIS. A further limitation is that the HIS Evaluation Framework and the questionnaires developed were in English. Thus, the pilot sample relied on participants who could read and understand English. For the most part, the pilot sample came from areas of nursing (n=4), medicine (n=3), and psychology (n=3). It would be useful for the questionnaire to be tested among a wider range of professional groups. Moreover, the MDS on the initial page of the survey was intended to capture the context and cultural data, but it may be assumed that a considerable amount of valuable information associated with the qualitative data is still missing [13]. This issue is being considered for future versions of the tool.

Finally, it was not possible to pilot level 5 (return on the HIS education investment) due to the time limitations of the project and the fact that the pilot modules were not fully developed at the time of the pilot.

The framework was implemented at “the First and Second Summer Program on Healthcare Improvement Science” course held by the University of Alicante in collaboration with the University of the West of Scotland in July 2016 and July 2017. This course, as it involved specific education in HIS, was used as part of the evaluation framework piloting. Its results will be used prospectively to keep improving the framework itself after collecting sufficient data from students from different fields and cultures, using several editions.

The evaluation framework has the potential to effectively identify strengths, weaknesses, and gaps in HIS education across Europe, as well as return on investment.

Investing in a better-educated professional staff regarding the scope of HIS could improve the quality of patient care [14] by building bridges between theory and practice and contributing to the development of an improvement culture in healthcare contexts.

## Figures and Tables

**Table 1.** Healthcare improvement science evaluation framework levels according to participants’ roles

	Student	Educator	Manager/ tutor	Manager/ professional
Level 1. reaction	V	V		
Level 2. learning	V	V		
Level 3. behavior/training transfer	V	V		
Level 4. results	V	V	V	V
Level 5. return on investment	V	V	V	V

## Supplementary materials

Supplement 1. Final report: Improvement Science Training for European Healthcare Workers (ISTEW) Workpackage Nº 10 Development of ISTEW evaluation framework.

Supplement 2. Audio recording of the abstract
