# Supplementary material for: Developing a framework for evaluating the impact of Healthcare Improvement Science Education across Europe: a qualitative study
Source: J Educ Eval Health Prof. 2017 Nov 29;14:28. doi: 10.3352/jeehp.2017.14.28 (PMC5801324; doi:10.3352/jeehp.2017.14.28)
Supplement: Supplementary file 1 — Supplement 1. Final report: Improvement Science Training for European Healthcare Workers (ISTEW) Workpackage Nº 10 Development of ISTEW evaluation framework. [file jeehp-14-28-suppl.pdf]

Improvement Science Training for European Healthcare Workers (ISTEW)

Workpackage Nº 10

DEVELOPMENT OF ISTEW EVALUATION FRAMEWORK

FINAL REPORT

INDEX :

- **WP10 OVERVIEW** **p. 5**
- **WP10 CONSENSUS DOCUMENT: PARTNERS CONTRIBUTION**
- **FRONT PAGES :**
  - ITALIAN FRONT PAGES p.16
  - POLISH FRONT PAGE p.19
  - SLOVENIAN FRONT PAGE p.27
  - SPANISH FRONT PAGES p.30
  - ROMANIAN FRONT PAGES p. 36
- **PILOTING** **p.44**
- **CONCLUSIONS** **p. 76**
- **LIMITATIONS** **p. 77**
- **FUTURE** **p. 78**

WP10 OVERVIEW

The WP10 is an implementation workpackage type. With a total duration of 20 months (From 5th month to 24th month) there are seven different partners participating, Scotland, UK, Italy, Poland, Romania, Slovenia and Spain. The Faculty of Health Sciences from the University of Alicante in Spain is the lead partner and the outputs expected are internal project documents, Skype meetings (including Minutes Documents) and also interim reports.

The main aims of this WP were to develop an evaluation framework by consensus for Healthcare Improvement Science Learning, identifying data appropriate allowing monitoring within and across all partner countries on an on-going basis, and also to enable the monitoring and evaluation of Healthcare Improvement Science education where it is implemented within and across countries on an ongoing basis.

To achieve those aims, **MDS and Case Study** were considered as the methods for WP10 in the ISTEW Project Proposal. **The Minimum Data Set** (MDS) was used to create the **Front Page** or first Data Collection Template in the questionnaire developed for the HIS Evaluation Framework. **The Case Study** was used to pilot the usability, understanding and content validation of the questionnaire developed.

Along the whole ongoing of WP10, two main processes were developed: first of all a “Developing Process for the HIS Evaluation Framework Design” and later a “Piloting Process for the HIS Evaluation Framework Content Validity and Usability”.

During the “Developing Process” the different levels of the HIS evaluation framework and also the specific participant samples were agreed by partners, as well as the kind of questions (open and closed questions with Likert Scale) and the language use.

On the other hand, in the “Piloting Process” each partner tested the contents understanding and the usability with real samples in their own contexts. Both processes were iterative ones and this is an important strength to be remarked.

The following chart agreed by all partners participating in WP10, exposes the different levels to be evaluated, correlated with the specific sample of participants that should answer in each level.

	STUDENT	EDUCATOR	MENTOR/TUTOR	MANAGER/PROFESSIONAL
LEVEL 1. REACTION	✓	✓		
LEVEL 2. LEARNING	✓	✓		
LEVEL 3. BEHAVIOUR/ TRAINING TRANSFER	✓	✓	✓	✓
LEVEL 4. RESULTS	✓	✓	✓	✓
LEVEL 5. RETURN OF INVESTMENT	✓	✓	✓	✓

The theoretical framework used is shown in the picture below where appears the Kirkpatrick's Model extracted from Parry et al. (2013).

Table 1. Kirkpatrick Framework for Evaluation and Application to Improvement Initiatives

Kirkpatrick Level	Evaluation	Applicability to Improvement Initiatives
1) Experience	What was the participants' experience?	Did the participants have an excellent experience working on the improvement project?
2) Learning	What did the participants learn?	Did the participants learn improvement methods and begin testing?
3) Process	Did they modify their behavior?	Did participants work differently and experience change in the process measures?
4) Outcome	Did the organization improve its performance?	Did the participants' organization improve its outcomes or performance?

During the developing process all partners agreed about the need of a last level related to "return on investment" (level 5) as it can be appreciated in the picture below where the table shows the adaptation of the Kirkpatrick evaluation framework levels to the ISTEW HIS evaluation framework as a first approach or step in the WP ongoing.

WP10 Case Study
(about HIS Learning Process):

Choose HIS Learning modules/courses in your country about these contents:
- *Systems thinking and process mapping.*
- *Models for improvement.*
- *Measurement for improvement.*
- *Communication and managing change.*

Global MDS (for the case):

HIS learning modules/courses characteristics/items/variables

- **Programme education level** (Undergraduate, Master Degree, PhD...)
- **Total number of hours and distribution** (theory, clinical placements, practice seminars...)
- **Programme on ...?** (Nursing, Medicine...)
- **Programme topic on...?** (community nursing, pediatrics, midwifery...)
- **How old is the course/module?**
- **Total number of students**
- **Is the course/module specifically/directly on HIS?**
- **Is the course/module related to HIS?** (referring to/or including HIS as a content)
- **Responsible professor/teacher profile/credentials.**
- **When did it start?** (the course/module)

To explore how knowledge (about HIS) is transferred into practice

To evaluate the impact of the module (about HIS)

Level 1. Reaction: *To evaluate participants initial response.*
- Did the participants like the training?
- What do they plan to do with what they learned?

- ***MDS Level 1:***
  - *Clinical placement setting (Where?)*
  - *Sex/Gender, Age*
  - *Previous HIS experience YES/NO*
  - *Previous healthcare placement experienced (in hours)*
  - ***Method Level 1:*** *Questionnaire (Check-list/structured questions with closed answers/with some open questions)*

Level 2. Learning: *To describe the knowledge participants gained and how they interpreted the learning.*
- What skills, knowledge, or attitudes changed after training? In which sense?

- ***MDS Level 2:***
  - *Which specific issues have been improved? (patient safety, patient satisfactions, healthcare quality aspects, infectious disease rates...)*
  - *Did you receive any specific theory/content about this issue previously? YES/NO*
  - ***Method Level 2:*** *Clinical competences evaluation tool (evaluation of knowledge, practice/skills and attitudes...) + open Questions/perceptions*

Level 3. Behavior / Training transfer: *To describe how participants have used the learning in the workplace*
- How participants change their behavior on-the-job based on what they learned?

- ***MDS Level 3:***
  - *Answered by the participant, did the participants act differently, according to the new knowledge? YES/NO*
  - *Is there any direct applicability of the improvement contents (taught in theory) to the context? YES/NO*
  - *Answered by the mentor, did the participants act differently, according to the new knowledge? YES/NO*
- ***Method Level 3:*** *Questionnaire to key informants (mentors) and questionnaire to participants/focus groups.*

Level 4. Results: *To describe how the participants use the learning in specific contexts.*
- How the change in behavior positively affect the organizations?

- ***MDS Level 4:***
  - *Have you noticed any possible learning gap about HIS? YES/NO*
  - *Has the change in behavior modified any indicator related to healthcare improvements? YES/NO*
  - ***Method Level 4:*** *Questionnaire to key informants (mentors) and questionnaire to healthcare professionals (clinical professionals, managers...)*

Level 5. Return on investment: *After the process*
- *Long term analysis*
- Was the training worth the cost?

- ***MDS Level 5:***
  - *Is there an ongoing learning process? YES/NO*
  - *Have the participant taken more courses on HIS after the process? YES/NO*
  - *Have the specific outputs (in this case) produced new knowledge? YES/NO*
  - *Have the participants continued developing the new skills? YES/NO*
  - *Have the participants seen these topics in other contexts? YES/NO*
- ***Method Level 5:*** *Questionnaire to participants (open/closed questions)*

**Draft Version 1**<br><br>**Dec 30, 2013**	**Spanish Team**	Spanish Team: First WP10 document was uploaded with all the key points all partners should knew about and consider before starting. It was just an Introduction to the WP10 and was based on our Kick-off presentation.
**Draft Version 2**<br><br>**Mar 4, 2014**	**Spanish Team**	Spanish Team uploaded the second draft version to start working deeply on both methods for development of ISTEW Evaluation Framework. It contained two specific protocols regarding every subgroup method so that every colleague would be informed about all stages to follow.
**Draft Version 3**<br><br>**Mar 27, 2014**	**Spanish Team**	Spanish Team uploaded a new draft for WP10 Specific Protocol (with both methods) and a WP10 Time Schedule.
**Draft Version 4**<br><br>**Mar 29, 2014**	**Rhoda Macrae**	**ETHICAL ISSUES**<br>*"I think it would be worth partners considering very carefully the case studies they select to include as as soon as we begin to 'talk to real people' we go over and above what we have ethical approval for (at least in the UK). In the UK going through health ethics takes a lot of time and effort and has the potential to delay th*<br>*e project. Perhaps this would be worth having a further conversation about once you have worked up some examples around the scope, type and depth of the information you hope to collect?"*
	**Spanish Team**	Adapting Suggestion in the last draft version for WP10 document with new changes included in red color.
**Draft Version 5**<br><br>**Apr 30, 2014**	**Spanish Team**	Basic information from the General WP10 Protocol was added to the Specific Protocol (next Draft Version) at the Introduction and Global Purpose. In step two (for both subgroups), all partners were asked to complete their healthcare contexts at different levels with: "Operational Definitions". It was also included specific colleagues' names at every specific subgroup protocol.
**Draft Version 6**<br><br>**Jun 6, 2014**	**Rhoda Macrae**	**DATA SOURCES**<br>*"The only comment I had was in relation to the data sources used within the kirkpatrick levels. It is suggested we talk to educators in level 1 - I think that educators what be a source of data at the level 3 point, as they would/could be part of the triangulation of data sources rather than at reaction level."*
	**Spanish Team**	Adapting Suggestion. New version included examples of the whole process. The Spanish team also uploaded the ppt presentation for

		Slovenia face-to-face meeting (including word template), MDS (word template), and case study example (word template -developed in Spanish) that was exposed and discussed at the meeting in Slovenia.
**Draft Version 7**<br><br>**Sept 24, 2014**	**Rhoda Macrae**	**DIAGRAM CONTENT**<br>*"In the diagram you ask the person evaluating to identifying HIS course of programme running, you list*<br>· *Identification of HIS challenges in clinical areas.*<br>· *Models for improvement.*<br>· *Measurement for improvement.*<br>· *Communication and managing change.*<br>*I notice that 3 of these are the planned titles of the modules to be developed and one is not. Is there a reason for choosing 'identification of HIS challenges in clinical areas' rather than systems thinking and process mapping? could systems thinking and process mapping also be included?"*
	**Marta Smoodis**	**QUESTION SUGGESTED**<br>*"The protocol looks very good. I would add a question of education, if they use of evidence-based and tracking innovations and transfer into clinical environment. Important is from where to obtain knowledge that they are up to date, which would suggest a question?"*
	**Katrina Ritters**	**STRUCTURE ISSUES AND FUTURE IMPACT**<br>*"On the questionnaires/analysis tools I wonder if I'm suffering from a misunderstanding. I thought our task was to develop tools that can be used by anyone who is putting on a course based on the modules we are developing; not for this project to do the actual analysis. I'm thinking about our discussion when we last spoke - that we won't actually have anything to evaluate until this particular ISTEW project is completed, the modules developed and delivered to students. It would be at that point that the institutions putting on the courses would do their evaluation - so that would be beyond the life of this particular project itself and/or dependent on future funding.*<br><br>*So the value of the work you are developing would be to produce a tool that individual institutions could use to demonstrate the value of the training they have provided.*<br><br>*If we were to have future funding, then I would think it would be a separate project to get people to use these tools with their students and centrally analyse the responses - but that would be several years into the future.*<br><br>*In terms of open ended/structured questions, I would have thought we could use a mix of Likert-scale type responses to standard questions with space for people to expand their answers if necessary."*
	**Spanish Team**	Adapting Suggestion.<br>The Spanish team updated the last documents published according to the last decisions taken about ISTEW Evaluation Framework on the last days of July.<br>Both documents on WP10 Minutes corresponded to the 25th of July teleconference attended by the following partners: UWS, Coventry University, Andy Carson-Stevens and the University of Alicante.<br>The Spanish team also worked on the new structure/model for WP10

		Evaluation Framework according to those last decisions and using the Kirkpatrick Framework for Evaluation and Application to Improvement Initiatives as a reference.
**Draft Version 8**<br><br>**Nov 10, 2014**	**Spanish Team**	The Spanish team worked on giving partners more details and options about the Methods suggested at each level and included them at the WP10 structure/model.
**Draft Version 9**<br><br>**Dec 3, 2014**	**Rhoda Macrae**	**CONTENT AND STRUCTURE SUGGESTIONS**<br>• *"That cover some of this kind of content"*<br>• *"Values?"*<br>• *"Notsure about Proper? Maybe Appropriate?"*<br>• *"Not sure about properly maybe Well? Clearly?"*<br>• *"Not sure about suitable maybe Optimised or designed for you / particpants to acquire knowledge"*<br>• *"Not sure aboutthe gramar perhaps 'Is there another methodology that could have been used to convey the knowledge more clearly?'"*<br>• *"Is this about reflective practice? Are you asking whether they are reflecting on the knowledge gained within the workplace?"*<br>• *"Not sure about changed for skills of knowledge would improved, increased for these be better?"*<br>• *"Has the training enhanced/improved any skills? If so can you describe*<br>• *"Has the training influenced your attitudes in any way? If so please describe"*<br>• *"Has the training enhanced/improved/increased your knowledge? If so can you describe"*<br>• *"Not sure waht this means"*<br>• *"Is this refering to their leanring processes or the training sturcutre, design/content?"*<br>• *"Not sure what you are trying to ask here, what do you want to know?"*<br>• *"This section is about finding out waht elements of the course and to what extent they applied the content in he workplace, yes?"*<br>• *"Not sure what you are trying to get at here"*<br>• *"This section wil just be for students and ex students, yes? Ae the educators likely to know whether and to waht extent they applied the material in practice?"*<br>• *"I think this is two questions in one so perahps Split it in two. Can you describe how your workplace context/culture enables the potential for HIS*<br>*Can you describe how your workpalce context/culture limits the potential for HIS"*<br>• *"I wonder if you are trying to get at the extent to which they actually put this in practice? If so maybe ask 'to what extent did you apply the content in the workplace"*<br>• *"Not sure what kind of data this question will produce – we really wantto get at what they applied, how they applied and why they applied it - yes? This comment applies to the Likert below. I wonder if we get them to describe how theys have used the learning in the workplace. The likerts might be better placed earlier for some of the level 1 and 2 questions"*<br>• *"Questions the encourage very specific examples of change would be good. So what changes have you seen? What different has*

		this made? Etc."<br>• *"Do we? Is this not about outcomes, the impact of transfer of learning? On the team/Ward/patient care?"*<br>• *"Are we asking the questions in this section of different people? I think we can ask ex students and key people around them? This is about triangluating the data, yes? If so we want to ask them to give specific examples of the impact/outcomes of the application of learning"*<br>• *"Notsure I understand these questions and not sure waht kind of data they will produce"*<br>• *"This has a very specific meaning in scotland and I don't think it is what you mean. Can you explain what you mean by community partnerships?"*<br>• *"Not sure if these questions are going to produce data on ROI perhaps someone else will offer ideas here, I will have to think it about it more"*
	**Spanish Team**	Adapting suggestion and also the Spanish team re-thought the methods they proposed as examples (in the structure uploaded) trying to find the less complex techniques/tools so that it would be easier for each partner to develop them at every level.<br>The Spanish team got an intermediate point between open and close questionnaire so that they could still get rich data but coming from not so open techniques, such as: semi-structured or even structured questionnaires and checklists.
**Draft Version 10**<br><br>**Dec 5, 2014**	**Rhoda Macrae**	**LEVEL 5 SUGGESTIONS**<br>*"I guess I am still not convinced that that this will enable us to get data at level 5. Perhaps other partners can contribute their views on this? Indeed I look forward to hearing the views of partners on the tool. I always find it useful to ask of every question within a research tool - 'is this going to produce the data that I need, that I will use, that will answer the question?' if the answer is no to any of these than the question either needs changed or taken out.*<br>*I am sure you have told us and my apologies for not paying attention - when do you anticipate partners to have completed the piloting of the tool?"*
	**Katrina Ritters**	**CONTENT AND STRUCTURED SUGGESTIONS**<br>*"I think you've done a great job Manuel in pulling together the theoretical framework into a series of practical questions relating to the modules we're now developing. We have a sound methodological framework for our questionnaire I feel, which will stand us in good stead in the future.*<br><br>*Also, from a practical point of view I think there are a number of things we could do to improve things still further from the perspective of the person filling in the form and also in terms of how we can learn from the results.*<br><br>*So, below I give a number of my initial reactions to the form - and am happy to help with re-drafting if you agree and if it would be helpful:*<br>*- I like the use of Lickert scales and think we could make even more use of them - for example to make them more specific and to add space for further clarification/information below some, if not all of them.*<br>*- I think we could make the questions more specific to the*

		knowledge, skills and attitude change we are trying to foster with the modules. For example, to be able to ask 'how far did the module help you develop your thinking on understanding processes for health improvement? - then to develop these themes as we work through to knowledge/behaviour transfer and results. Of course, we won't be in a position to refine the questionnaire until the modules are further developed, but this sort of approach may well make the questionnaire more useful.<br>- *It's good to make the questionnaire as easy for the user to fill in as possible. It may help if we make the initial section - about the module, course etc - standard and filled in by the tutor or as part of the pre-printed form, so the respondent only has to fill in those parts of the questionnaire that are relevant to them.*<br>- *At the moment we've pulled together all of Kirkpatrick's evaluation stages into one questionnaire, but when it comes to using it in practice, it may make more sense to have a different questionnaire for those people at each stage of their learning journey - ie - perhaps one for those who have just started the course - why have they done it? What are they hoping it will do for them? Does it look as if their expectations are being met? Then another one at the end of the course - what have they learned? How do they expect to be able to apply it? And another a year or so after people have finished - what has changed? At all stages, how could the course be improved to make it more useful/relevant/impactful?*<br>- *Maybe we should think also about how the questionnaires will be analysed. Is there relevant software out there that would be acceptable to our universities ethics people that would pull all the results together into a standard format and save on administrative time? (our University uses Bristol Online Survey)*<br>- *As is only to be expected, there are one or two places where the nuance of language could be improved - Rhoda and I can help with this; the main thing at the moment though I think is for us to be sure that we've covered the ground in terms of what we want to know, from whom and at what point on their learning journey."*
	**Barbara O'Donnell**	**TO ADAPT LANGUAGE STYLE TO STUDENTS**<br>*"I think my own view is around some of the language we are using. There are a lot of very academic terms and, bearing in mind, many of the students are likely to be healthcare workers, and many at undergraduate level, I think we need to consider some of the words we have used. For example: curricular objectives may be as well to read course objectives, methodology and didactic could also present a challenge for this type of student. i think the language needs to reflect the student's understanding rather than ours."*
	**Julita Sansoni**	**GENERAL DOUBTS**<br>*"I am a bit confused because I do miss if you/we want to create a new tool that measure something or if you want to collect opinions to interpret by qualitative lens.*<br>*In the first case, I personally would state the questions differently (in a more measurable way). If it is the second, I need some clarification on the general aim/objective of what we want to do.*<br><br>*Are we intending to measure the efficacy of education that we have taken into consideration (WP6) or is this an instrument for the future education (modules that will be produced by our project)?"*

	**Spanish Team**	Adapting suggestions and also the Spanish team worked further more regarding the techniques to be used at the case study protocol.
**Draft Version 11**<br><br>**Dec 22, 2014**	**Katrina Ritters**	**CONTENT AND STRUCTURE SUGGESTIONS**<br>• *"In this section, I would suggest pre-populated answers provided by the tutor before it goes to the student - or leave this section out of the questionnaire that goes to the student and attach the information to their answers in some other way."*<br>• *"This will need more detail but I appreciate this cannot happen until the modules themselves are more fully developed."*<br>• *"Hopefully, these categories reflect categories appropriate for the UK, but they may not work for all partners."*<br>• *"Don't ask participants to fill this in - either design as part of the form or ask the tutor to fill in this part."*<br>• *"Is this relevant?"*<br>• *"Use question inserted above to get this detail"*<br>• *"This might be too onerous for people to fill in - and complicated to analyse."*<br>• *"The specifics here might be more relevant if they relate to the stated course objectives/content. Either that or keep them very vague, as indicated"*<br>• *"Use lickert scale instead of yes/no"*<br>• *"Lickert scale"*<br>• *"Values?"*<br>• *"Good question"*<br>• *"Values?"*<br>• *"Lickert scale"*<br>• *"Lickert scale - add in not applicable for those people not undertaking other training"*<br>• *"I ickert"*<br>• *"possibly supplement with - was it easy to understand?"*<br>• *"Use Lickert scales"*<br>• *"Once we have the course outlines, it might be worth breaking down the key elements of the course and asking whether the information was easy to understand/presented clearly"*<br>• *"I would leave this question out - cover it in other ways such as in the above question. Or as a supplementary to the question above, just ask what could improve the course design or presentation.."*<br>• *"See above comment - could lose this question"*<br>• *"Is this about reflective practice? Are you asking whether they are reflecting on the knowledge gained within the workplace?"*<br>• *"Not sure about changed for skills of knowledge would improved, increased for these be better?"*<br>• *"Think this may already be covered – above"*<br>• *"Think this question needs more detail - eg in relation to each element of the course - could be patient safety; consistency; teamwork - whatever the course has been focused on. Would be interesting to know to what extent it has improved in each area - use lickert scales for this."*<br>• *"Not sure waht this means"*<br>• *"I would delete this question - already covered by above and too vague to be much use"*<br>• *"Is this refering to their leanring processes or the training sturcutre, design/content?"*<br>• *"I would delete this question - already covered by above and too vague to be much use."*

		• *"Not sure what you are trying to ask here, what do you want to know?"*<br>• *"I would delete this question - if it is asking about changes in others' behaviour that may have nothing to do with the course."*<br>• *"Not sure what kind of data this question will produce – we really wantto get at what they applied, how they applied and why they applied it - yes? This comment applies to the Likert below. I wonder if we get them to describe how theys have used the learning in the workplace. The likerts might be better placed earlier for some of the level 1 and 2 questions"*<br>• *"I would delete this question - already covered by above and too vague to be much use"*<br>• *"This section is about finding out waht elements of the course and to what extent they applied the content in he workplace, yes?"*<br>• *"Not sure what you are trying to get at here."*<br>• *"This section wil just be for students and ex students, yes? Ae the educators likely to know whether and to waht extent they applied the material in practice?"*<br>• *"I think this is two questions in one so perahps Split it in two. Can you describe how your workplace context/culture enables the potential for HIS. Can you describe how yourworkpalce context/culture limits the potential for HIS."*<br>• *"Are we asking the questions in this section of different people? I think we can ask ex students and key people around them? This is about triangluating the data, yes? If so we want to ask them to give specific examples of the impact/outcomes of the application of learning"*<br>• *"Notsure I understand these questions and not sure waht kind of data they will produce"*<br>• *"This has a very specific meaning in scotland and I don't think it is what you mean. Can you explain what you mean by community partnerships?"*<br>• *"Not sure if these questions are going to produce data on ROI perhaps someone else will offer ideas here, I will have to think it about it more."*
	**Spanish Team**	Adapting suggestion and also adapting the document to some documents the Spanish team had used to modify our last WP10 Protocol according to our last skype meeting (all of them posted on Basecamp). All changes were in red colour.
**Draft Version 12**<br><br>**Dec 29, 2014**	**Rhoda Macrae**	**CONTENT AND STRUCTURE SUGGESTIONS**<br>*"This is the tool that we will use to evaluate the impact of the course and to explore how knowledge is transferred / applied in practice. The tool is both for capturing impact of learning (Kirkpatrick level 1-4 plus ROI) and MDS data. Yes?*<br>*The tool will be researcher administered during the pilot, however going forward will this be a self complete tool? the type of instructions and signposting given throughout the tool will depend on whether whether it is a self complete or administered tool. The tool will also need an introduction, stating its purpose i.e to measure the impact of HIS courses or courses with elements of HIS . Will need to state who it is for, which sections apply to only educators, only students etc. and signpost whoever is completing it around the tool e.g are there sections they jump to? Etc..*<br><br>*I piloted this on my daughter who is training to be a nurse, although*

		she doesn't really know what HIS is she was able to give me feedback on the tool – we pretended it was about the course she is doing now, it was helpful as it highlighted the questions that didn't make sense to her - she was very forthcoming!<br>• *For students only?*<br>• *A variety of terminology is being used – training, programme, learning and course. I think we need to stick with one, probably course*<br>• *Not sure what data this is going to yeild. Not sure how this taps in to their reaction or what you are trying to ask*<br>• *However they will only know what they know and may not be equipped to answer this.*<br>• *Could this be a Likert? Please indicate how relevant the course objectives are to your work at present?*<br>• *If they are current students they might not know yet*<br>• *Could this be a Likert asking them to rate the learning experience?*<br>• *Not sure what kind of data this is goingto yeild. Is this asking about the extent to which participation enhanced the learning process?*<br>• *Not sure what kind of data this is going to yeild. Is this asking about whether there was enough opportunties for students to particpate?*<br>• *Are you asking what helped them to engage in the learning process?*<br>• *Is this asking about whether and in what they think this course will be useful in the future or how they think they will apply what they have learnt in the future?*<br>• *Great question*<br>• *Because the general information asks about MDS for the case section one needs to focus on reactions, did they like it? Why? Did it meet their expectations? Why? What did they like the most? Why? What do they plan to do with what they have learnt so far? I wonder if section 1 can be made more concise, perhaps ask fewer questions? Some of the questions are very similar so perhaps there is a way of combining some so they capture great data about their reaction.*<br>• *Would the scale be very useful – not at all useful? Also, useful – in terms of what, this section is on what they have learnt*<br>• *Not sure ifthis is going to capture what they have learnt*<br>• *Not sure what data this is going to yeild?*<br>• *They only know wahtthey know, they might not know wahtthe intentions were*<br>• *There may have been more tan one method, perhaps this is about asking whiich methods helped them learn most?*<br>• *Not sure what this question is asking*<br>• *This question could lead on from or be combined with the one about methods*<br>• *Perhaps this is about aking whether and in what way did these resources enhance their learning*<br>• *Is this asking whethr they are satisified about how much and what they learnt?*<br>• *Not sure what this is going to tell us*<br>• *This is a yes/no question. Perhaps the question is about asking them to descibe how waht hey have learnt has relevance for their practice.*<br>• *This might need rephrasing. They might tick all or some, ifthe tick all we won't know wherethe most knowledge gain has been. Maybe break this down tand get them o rate or describe where they have had the most knowledge gain.*<br>• *It isn't clear what is being asked*

		Section 2 is about increase in knowledge or capability as a result of participating in the course. What skills, attitudes, knowledge have changed / increased after the course. What do they know now that they didn't know before, what have they learnt? What have they learnt most about? Describe Why? Were their learning expectations met? Did they enhance their skills? In what way? Have their attitudes changed? In what way? I think some of the questions could be combined/rephrased to produce a more concise and focused section. Also need to bear in mind the MDS at this level, do the questions encompass these?<br><br>• *This is a yes/no question needsto ask to what extent have they applied…..*<br><br>• *I don't understand this question. Maybe a question on what aspects of the course have they applied in practice? Used some of the knowledge, the skills? Has the course changed there attidtudes about anything? Increased their understanding about HIS as applied in practice?*<br>• *Is this asking about alignment, relevance?*<br>• *See comment about Q1*<br>• *This is potentially interesting if we know what proved difficult and why? So we need to realte this to the course, waht can the educators do to support students over come barriers to application?*<br>• *Not sure waht you are trying to ask here*<br><br>*We need to get them to describe specifically how they have used the learning in the workplace. What they are doing differently now as a result of being on the course. These questions need to be also asked of someone in the workplace, a supervisor, manager, mentor as this is about transfer"*<br><br>*"For students and educators? For mentors, supervisors, managers in the workplace of the student?*<br>*Need to describe the impact of the changed behavior, so if they are doing things differently (which if they are they will have described the previous section) what difference is this making to them, their colleagues, the workplace, the clients? Can they describe/evidence this?"*<br><br>*"This section is all about impact – whether and to what extent the learning has impacted on the individuals practice, and the whether and to what extent this has impacted more widely in the workplace. Asking for specific examples of impact will be useful . Now we have introduced …….. this is the difference it makes to………………………….it makes a difference in this way…………."*<br><br><br>*"There is something about the timing of each of these sections that we haven't made clear but we have discussed and it is in the framework. Reaction is immediate, learning is within 3 months, behavior within 3-6 months, impact within 6-9 months, ROI is much later. Was the training worth the cost? ROI for who? the person that did the course? probably not, so who are we asking about this? I think we need to discuss this more, perhaps Andy can help us? Not sure about questions 1 and 2. I think this is about ongoing learning processes, have the kept developing their skills and knowledge?"*<br><br>• *"Not sure about this question, one might expect it of some courses*

		but not others?"
	**Julita Sansoni**	**CONTENT AND STRUCTURE SUGGESTIONS**<br>• *"Please give an example on how you used in your work at present"*<br>• *"This can be a question yes/not . Have you learned new topics?"*<br>• *"What type of method of teaching was used? Active involving /Ex cathedra"*<br>• *"It seems to be too subjective"*<br>• *"Plese give an example of what you think to have learnt most"*<br>• *"Could you describe the process that you have personally lived during the course?"*<br>• *"Do you think to have had the necessary background"*<br>• *"When I'm working I think about the knowledge that I have gained YES/NO/SOMETIME"*<br>• *"I think that we have to ask three separate questions."*<br>• *"What kind of change or improvement have you been able to use in your working place"*<br>• *"Separate questions."*<br>• *"Have you been able to share with your collegue in your working place the content/Knowledgereceived during the course?"*<br>• *"Ask for an example."*
	**Spanish Team**	Adapting suggestions and modifying all levels, especially level 5. The Spanish team changed the Focus Group topic or issue to discuss including more specific questions at Level 5 that could describe and let them know how that "return" happened.
**First FRONT PAGE Draft**<br><br>**Feb 17, 2015**	**Spanish Team**	Spanish Team uploaded WP10 FRONT PAGE of the WP10 Evaluation Framework Questionnaire/Tool with the MDS (items/variables) regarding the "Case" (considered as the course/module selected by each partner) every partner had to choose. After that, every partner had to freely select their "Case" (module/subject/course with HIS elements in their own country/context/university) and send it back to the Spanish team. **Deadline: 27th of February, 2015**.<br><br>Spanish Team included "Instructions" to complete it. The document included a "General Introduction" to the WP10 Questionnaire/Tool and at the end there was another point called "Next Instructions" which is an introduction to the next steps they were going to take and it contained the chart/scheme suggested by Rhoda and its explanation.
**Answer to Draft Version 12**<br><br>**Dec 29, 2014**<br><br>**Posted on Feb 20, 2015**	**Marta Smodis**	**CONTENT AND STRUCTURE SUGGESTIONS**<br>• *"HIS, MDS -– all abbreviation had to be explained"*<br><br>• *"Globaly or in one's own country or school"*
**Draft Version 13**<br><br>**Second FRONT PAGE Draft**<br><br>**Feb 23,**	**Spaish Team**	Spanish Team uploaded INDIVIDUAL QUESTIONNAIRES corresponding to the different PARTICIPANTS and at different LEVELS. Spanish Team also included Slovenian Team comments.<br><br>The 30th of April Spanish Team uploaded WP10 FINAL REPORT 1st DRAFT VERSION.<br><br>The 1th of May Spanish Team uploaded WP10 FINAL REPORT 1st DRAFT VERSION with last colleagues piloting results added.

**2015**		The 7th of May Spanish Team uploaded WP10 FINAL REPORT 1st DRAFT VERSION 7/5/15 with last colleagues piloting results added.
	**Monika Baryła-Matejczuk**	The 9th of March Polish Team sent FRONT PAGE MDS by two students of nursing and one psychology student.
	**Marta Smodis**	The 10th of March Slovenian Team sent FRONT PAGE MDS.<br><br>The 29th of April Slovenian Team sent their piloting results.<br><br>The 30th of April Slovenian Team sent the rest of their piloting.
	**Julita Sansoni**	The 12th of March Italian Team sent FRONT PAGE MDS.
	**Laura Smochina**	The 31th of April Romanian Team sent FRONT PAGE MDS.<br><br>The 30th of April Romanian Team sent their piloting results.<br><br>The 4th of May Romanian Team sent the rest of their piloting.
	**Magdalena Glowacka**	The 6th of May Polish Team sent their piloting results.<br><br>The 18th of May Polish Team sent the rest of their piloting results.
	**Katrina Ritters**	The 14th of May Coventry Team sent their piloting results.
	**Joanna Girzelska**	The 18th of May Polish Team sent the rest of their piloting results.
**Draft Version 14**<br>**May 19, 2015**	**Julita Sansoni**	The 9th of July Italian Team sent their piloting results.
	**Barbara O'Donell**	The 13th of July UWS Team sent their piloting results.
	**Laura Smochina**	The 15th of July Romanian Team sent their piloting conclusions.
**Draft Version 15**<br>**July 22, 2015**	**Spanish Team**	Spanish Team uploaded WP10 FINAL REPORT 15st DRAFT VERSION with last colleagues piloting results and comments added.<br><br>The 31th of July Spanish Team uploaded the WP10 EXECUTIVE SUMMARY as a summary of the whole process.
**Draft Version 16**<br>**Oct 07, 2015**	**Spanish Team**	After ISTEW Conference, Spanish Team uploaded WP10 FINAL REPORT 16st DRAFT VERSION with last colleagues suggestions.

WP10-HIS Learning Evaluation Framework FRONT PAGE:

ITALIAN FRONT PAGE:

GENERAL INFORMATION ABOUT HIS MODULE/COURSE (TO BE FILLED BY TUTORS/MENTORS)

***Please, specify the module/course title. Choose one of these two options:**

1. **Healthcare Improvement Science module/course (those using the SPECIFIC concept "Healthcare Improvement Science"):**

   ____

2. **Other modules/courses (those not using the SPECIFIC concept "Healthcare Improvement Science"):**

   Quality of Care and Safety

   2.1 If you have chosen this option, please tick the content/s included in that module/course (indirectly related to HIS):

   - □ Systems thinking and process mapping.
   - × Models for improvement.
   - × Measurement for improvement.
   - × Communication and managing change.

***Please, specify the module/course characteristics:**

3. **Programme education level:**
   - □ PhD/DNP Programme
   - × Master Degree
   - □ Postgraduate Programme
   - □ Graduate Programme
   - □ Undergraduate Programme
   - □ Professional Education
   - □ Specialization Programme
   - □ Others:

4. **Total number of hours and distribution:**
   - □ Theoretical Sessions: 50 hours
   - □ Clinical/Other Placement:
   - □ Practice Seminars: 2 seminars of 16 hours

- ◻ Lab Seminars:
- ◻ Others:

*Total number of hours: 82 hours

5. **Course/Module programme discipline:**
   - × Nursing
   - ◻ Medicine
   - ◻ Social work
   - ◻ Psychology
   - ◻ Physiotherapy
   - ◻ Occupational Therapy
   - ◻ Pharmacology
   - ◻ Nutrition
   - ◻ Others

6. **Course/Module programme´s topic/specialization** (e.g. community nursing): University Second level Degree (future Nurse managers)

7. **Course/module organizing institution**: Sapienza University of Rome

8. **Course/module settings:**
   - × Theoretical Education setting:
   - × Practice Education setting:

9. **Course/module edition number**: every year since around 2000

10. **Course/module total number of students**: 60 per class

11. **Course/module responsible professors/teachers**: Academic professors, adjunt professors and Staff (Services) personnell

12. **Course/module professors´/teachers´ education background on HIS** (specify the title):
   - ◻ PhD/DNP Programme:
   - ◻ Master Degree:
   - ◻ Postgraduate Programme:
   - ◻ Graduate Programme:
   - ◻ Undergraduate Programme:
   - ◻ Professional Education:
   - ◻ Specialization Programme:
   - × Research experience on HIS:
   - × Others: Faculties have followed specific Courses (as Continuing Education courses) that can be governamental, University or Service based, We have corses preparing people as experts in Quality, expert in Accreditation etc.…We do not offer education (Master, Degree or PhD) in HIS. (i.e.: it is possible to get PhD in Nursing focusing dissertation on Quality (in future could be HIS) as well as other subjects but your title will be PhD in Nursing.

GENERAL INFORMATION ABOUT HIS STUDENTS/ALUMNI (TO BE FILLED BY STUDENTS/ALUMNI)

13. Please indicate your professional role:

- □ Healthcare assistant
- □ Registered Nurse (RN)
- □ Specialized Nurse (including Nurse Practitioner)
- □ General Practitioner (GP)
- □ Specialized Medical Doctor (MD)
- □ Managerial/senior consultant
- □ Administrative
- □ Social worker
- □ Psychologist
- □ Physiotherapist
- □ Occupational Therapist
- □ Pharmacologist
- □ Nutritionist
- × Others MNSc Student

14. Please indicate your academic level:

- □ PhD/DNP :
- □ Master Degree:
- □ Postgraduate:
- × Graduate:
- □ Undergraduate:
- □ Specialization:
- □ Others:

15. Sex:

15.1 Man
15.2 Woman

16. **Age**: 40

17. **Previous HIS experience**:

17.1 No
17.2 Yes

- □ Experience in hours: 30
- □ Type of education programme: Professional Master
- □ Name of the programme: Master in Coordinamento (Head Nurs Professional Master)

POLISH FRONT PAGE.1 :

GENERAL INFORMATION ABOUT HIS MODULE/COURSE (TO BE FILLED BY TUTORS/MENTORS)

***Please, specify the module/course title. Choose one of these two options:**

1. **Healthcare Improvement Science module/course (those using the SPECIFIC concept "Healthcare Improvement Science"):**

2. **Other modules/courses (those not using the SPECIFIC concept "Healthcare Improvement Science"): Psychology**

   2.1 If you have chosen this option, please tick the content/s included in that module/course (indirectly related to HIS):

   ×Systems thinking and process mapping.
   ×Models for improvement.
   ×Measurement for improvement.
   ×Communication and managing change.

***Please, specify the module/course characteristics:**

3. **Programme education level:**
   - ☐ PhD/DNP Programme
   - ☐ Master Degree
   - ☐ Postgraduate Programme
   - ☐ Graduate Programme
   - × Undergraduate Programme
   - ☐ Professional Education
   - ☐ Specialization Programme
   - ☐ Others:

4. **Total number of hours and distribution:**

   ×Theoretical Sessions: 1105
   ×Clinical/Other Placement: 130
   ×Practice Seminars: 1015
   ×Lab Seminars: 380
   ×Others: 80
   *Total number of hours: 2710

5. **Course/Module programme discipline:**
   - ☐ Nursing
   - ☐ Medicine
   - ☐ Social work

× Psychology
□ Physiotherapy
□ Occupational Therapy
□ Pharmacology
□ Nutrition
□ Others

6. **Course/Module programme´s topic/specialization** (e.g. community nursing):
   Prophylaxis and Counselling

7. **Course/module organizing institution:**
   University of Economics and Innovation

8. **Course/module settings:**
   □ Theoretical Education setting:
   □ Practice Education setting:

9. **Course/module edition number:**

10. **Course/module total number of students:**

11. **Course/module responsible professors/teachers:**

12. **Course/module professors´/teachers´ education background on HIS (specify the title):**
   □ PhD/DNP Programme:
   □ Master Degree:
   □ Postgraduate Programme:
   □ Graduate Programme:
   □ Undergraduate Programme:
   □ Professional Education:
   □ Specialization Programme:
   □ Research experience on HIS:
   □ Others:

GENERAL INFORMATION ABOUT HIS STUDENTS/ALUMNI (TO BE FILLED BY STUDENTS/ALUMNI)

13. **Please indicate your professional role:**

   □ Healthcare assistant
   □ Registered Nurse (RN)
   □ Specialized Nurse (including Nurse Practitioner)
   □ General Practitioner (GP)
   □ Specialized Medical Doctor (MD)
   □ Managerial/senior consultant
   □ Administrative
   □ Social worker
   × Psychologist

- □ Physiotherapist
- □ Occupational Therapist
- □ Pharmacologist
- □ Nutritionist
- □ Others:

14. Please indicate your academic level:

- □ PhD/DNP :
- × Master Degree:
- □ Postgraduate:
- □ Graduate:
- □ Undergraduate:
- □ Specialization:
- □ Others:

15. Sex:

15.1 × Man

16. **Age:** 24

17. Previous HIS experience:

17.1 × No

17.2 Yes

- □ Experience in hours:
- □ Type of education programme:
- □ Name of the programme:

POLISH FRONT PAGE.2 :

GENERAL INFORMATION ABOUT HIS MODULE/COURSE (TO BE FILLED BY TUTORS/MENTORS)

***Please, specify the module/course title. Choose one of these two options:**

1. **Healthcare Improvement Science module/course (those using the SPECIFIC concept "Healthcare Improvement Science"):**

2. **Other modules/courses (those not using the SPECIFIC concept "Healthcare Improvement Science"):**

Nursing

2.1 If you have chosen this option, please tick the content/s included in that module/course (indirectly related to HIS):

- □ Systems thinking and process mapping.
- □ Models for improvement.
- □ Measurement for improvement.
- x Communication and managing change.

***Please, specify the module/course characteristics:**

3. Programme education level:

- □ PhD/DNP Programme
- □ Master Degree
- □ Postgraduate Programme
- □ Graduate Programme
- x Undergraduate Programme
- □ Professional Education
- □ Specialization Programme
- □ Others:

4. Total number of hours and distribution:

- x Theoretical Sessions:
- □ Clinical/Other Placement:
- □ Practice Seminars:
- □ Lab Seminars:
- □ Others: 15

*Total number of hours: 30

5. Course/Module programme discipline:

- x Nursing
- □ Medicine
- □ Social work
- □ Psychology
- □ Physiotherapy
- □ Occupational Therapy
- □ Pharmacology
- □ Nutrition
- □ Others

6. Course/Module programme´s topic/specialization (e.g. community nursing):

Interpersonal communication

7. **Course/module organizing institution:** University of Economics and Innovation - The Faculty of Pedagogy and Psychology

8. Course/module settings:

- x Theoretical Education setting: university
- □ Practice Education setting:

9. **Course/module edition number**: 6

10. **Course/module total number of students**: 95

11. **Course/module responsible professors/teachers:** 1

12. **Course/module professors´/teachers´ education background on HIS (specify the title):**
   - ◻ PhD/DNP Programme: Doctor of Medicine
   - ◻ Master Degree: Graduated from the Faculty of Philosophy and Sociology
   - ◻ Postgraduate Programme:
   - ◻ Graduate Programme:
   - ◻ Undergraduate Programme:
   - ◻ Professional Education:
   - ◻ Specialization Programme:
   - ◻ Research experience on HIS: The author and co-author of several scientific papers in the field of interpersonal communication, psycho, and psychosocial aspects of health and medical care.
   - ◻ Others: Scientific and professional issues involved in interpersonal and social communication in medicine and health sciences, and particularly the media of social communication and information and communication technology (the Internet).

GENERAL INFORMATION ABOUT HIS STUDENTS/ALUMNI **(TO BE FILLED BY STUDENTS/ALUMNI)**

13. **Please indicate your professional role:**

   - ◻ Healthcare assistant
   - x Registered Nurse (RN)
   - ◻ Specialized Nurse (including Nurse Practitioner)
   - ◻ General Practitioner (GP)
   - ◻ Specialized Medical Doctor (MD)
   - ◻ Managerial/senior consultant
   - ◻ Administrative
   - ◻ Social worker
   - ◻ Psychologist
   - ◻ Physiotherapist
   - ◻ Occupational Therapist
   - ◻ Pharmacologist
   - ◻ Nutritionist
   - ◻ Others:

14. **Please indicate your academic level:**

   - ◻ PhD/DNP :

- ◻ Master Degree:
- ◻ Postgraduate:
- ◻ Graduate:
- x Undergraduate:
- ◻ Specialization:
- ◻ Others:

15. Sex:

15.1 Man

x Woman

16. **Age:** 32

17. Previous HIS experience:

17.1 No

17.2 Yes x

- ◻ Experience in hours:
- ◻ Type of education programme:
- ◻ Name of the programme: public health programme

POLISH FRONT PAGE.3 :

GENERAL INFORMATION ABOUT HIS MODULE/COURSE (TO BE FILLED BY TUTORS/MENTORS)

***Please, specify the module/course title. Choose one of these two options:**

1. **Healthcare Improvement Science module/course (those using the SPECIFIC concept "Healthcare Improvement Science"):**

2. **Other modules/courses (those not using the SPECIFIC concept "Healthcare Improvement Science"):**

Nursing

2.1 If you have chosen this option, please tick the content/s included in that module/course (indirectly related to HIS):

- ◻ Systems thinking and process mapping.
- ◻ Models for improvement.
- ◻ Measurement for improvement.
- x Communication and managing change.

***Please, specify the module/course characteristics:**

3. **Programme education level:**
   - ◻ PhD/DNP Programme
   - x Master Degree
   - ◻ Postgraduate Programme
   - ◻ Graduate Programme
   - ◻ Undergraduate Programme
   - ◻ Professional Education
   - ◻ Specialization Programme
   - ◻ Others:

4. **Total number of hours and distribution:**
   - x Theoretical Sessions: 27
   - x Clinical/Other Placement: 40
   - x Practice Seminars: 10
   - ◻ Lab Seminars:
   - ◻ Others:

   *Total number of hours: 77

5. **Course/Module programme discipline:**
   - x Nursing
   - ◻ Medicine
   - ◻ Social work
   - ◻ Psychology
   - ◻ Physiotherapy
   - ◻ Occupational Therapy
   - ◻ Pharmacology
   - ◻ Nutrition
   - ◻ Others

6. **Course/Module programme´s topic/specialization** (e.g. community nursing): Nursing management

7. **Course/module organizing institution:** University of Economics and Innovation - The Faculty of Pedagogy and Psychology

8. **Course/module settings:**
   - x Theoretical Education setting: University
   - x Practice Education setting: Hospital

9. **Course/module edition number:**

10. **Course/module total number of students**: 95

11. **Course/module responsible professors/teachers:** 2

12. **Course/module professors´/teachers´ education background on HIS (specify the title):**

X PhD/DNP Programme:
□ Master Degree:
□ Postgraduate Programme:
□ Graduate Programme:
□ Undergraduate Programme:
□ Professional Education:
□ Specialization Programme:
□ Research experience on HIS:
□ Others:

GENERAL INFORMATION ABOUT HIS STUDENTS/ALUMNI (TO BE FILLED BY STUDENTS/ALUMNI)

13. Please indicate your professional role:

□ Healthcare assistant
x Registered Nurse (RN)
□ Specialized Nurse (including Nurse Practitioner)
□ General Practitioner (GP)
□ Specialized Medical Doctor (MD)
□ Managerial/senior consultant
□ Administrative
□ Social worker
□ Psychologist
□ Physiotherapist
□ Occupational Therapist
□ Pharmacologist
□ Nutritionist
□ Others:

14. Please indicate your academic level:

□ PhD/DNP :
□ Master Degree:
□ Postgraduate:
x Graduate:
□ Undergraduate:
□ Specialization:
□ Others:

15. Sex:
15.1 Man
x Woman

16. Age: 31

17. Previous HIS experience:

17.1 No
17.2 Yes

- □ Experience in hours:
- □ Type of education programme:
- □ Name of the programme:

SLOVENIAN FRONT PAGE:

GENERAL INFORMATION ABOUT HIS MODULE/COURSE (TO BE FILLED BY TUTORS/MENTORS)

***Please, specify the module/course title. Choose one of these two options:**

1. **Healthcare Improvement Science module/course (those using the SPECIFIC concept "Healthcare Improvement Science"):**

Basic of Management and Quality in Health Care and Nursing Care

2. **Other modules/courses (those not using the SPECIFIC concept "Healthcare Improvement Science"):**
   - Design of Clinical Microsystems and Continuous Quality and Patient Safety Improvement
   - Patient Safety
   - Leadership in Nursing

2.1 If you have chosen this option, please tick the content/s included in that module/course (indirectly related to HIS):

- □ Systems thinking and process mapping.
- □ Models for improvement.
- X Measurement for improvement.
- □ Communication and managing change.

***Please, specify the module/course characteristics:**

3. **Programme education level:**
   - □ PhD/DNP Programme
   - X Master Degree
   - □ Postgraduate Programme
   - □ Graduate Programme
   - □ Undergraduate Programme
   - □ Professional Education
   - □ Specialization Programme

- ☐ Others:

4. **Total number of hours and distribution:**
   - X Theoretical Sessions: 40
   - ☐ Clinical/Other Placement: ______________________________
   - X Practice Seminars: 40
   - ☐ Lab Seminars: ____
   - X Others: CONTACT HOURS – EXERCISES : 20
     INDIVIDUAL STUDENT WORK: 150

   *Total number of hours: 250

5. **Course/Module programme discipline:**
   - X Nursing
   - ☐ Medicine
   - ☐ Social work
   - ☐ Psychology
   - ☐ Physiotherapy
   - ☐ Occupational Therapy
   - ☐ Pharmacology
   - ☐ Nutrition
   - ☐ Others

6. **Course/Module programme´s topic/specialization** (e.g. community nursing):

7. **Course/module organizing institution**: FHCJ

8. **Course/module settings:**
   - X Theoretical Education setting: FHCJ
   - ☐ Practice Education setting:

9. **Course/module edition number**:

10. **Course/module total number of students**: 13

11. **Course/module responsible professors/teachers:** 2

12. **Course/module professors´/teachers´ education background on HIS** (specify the title):
    - X PhD/DNP Programme:
    - ☐ Master Degree:
    - ☐ Postgraduate Programme:
    - ☐ Graduate Programme:
    - ☐ Undergraduate Programme:
    - ☐ Professional Education:
    - ☐ Specialization Programme:
    - ☐ Research experience on HIS:

- □ Others:

GENERAL INFORMATION ABOUT HIS STUDENTS/ALUMNI (TO BE FILLED BY STUDENTS/ALUMNI)

13. Please indicate your professional role:

- □ Healthcare assistant
- X Registered Nurse (RN)
- □ Specialized Nurse (including Nurse Practitioner)
- □ General Practitioner (GP)
- □ Specialized Medical Doctor (MD)
- □ Managerial/senior consultant
- □ Administrative
- □ Social worker
- □ Psychologist
- □ Physiotherapist
- □ Occupational Therapist
- □ Pharmacologist
- □ Nutritionist
- □ Others:

14. Please indicate your academic level:

- □ PhD/DNP :
- □ Master Degree:
- □ Postgraduate:
- X Graduate:
- □ Undergraduate:
- □ Specialization:
- □ Others:

15. Sex:

15.1 Man
15.2 Woman

16. Age: 39

17. Previous HIS experience:

17.1 No
17.2 Yes

- □ Experience in hours:
- □ Type of education programme:
- □ Name of the programme:

SPANISH FRONT PAGE. 1 :

GENERAL INFORMATION ABOUT HIS MODULE/COURSE (TO BE FILLED BY TUTORS/MENTORS)

***Please, specify the module/course title. Choose one of these two options:**

1. **Healthcare Improvement Science module/course (those using the SPECIFIC concept "Healthcare Improvement Science"):**

2. **Other modules/courses (those not using the SPECIFIC concept "Healthcare Improvement Science"):**

   Nursing

   2.1 If you have chosen this option, please tick the content/s included in that module/course (indirectly related to HIS):

   x Systems thinking and process mapping.

   x Models for improvement.

   x Measurement for improvement.

   x Communication and managing change.

***Please, specify the module/course characteristics:**

3. **Programme education level:**
   - □ PhD/DNP Programme
   - □ Master Degree
   - □ Postgraduate Programme
   - □ Graduate Programme
   - x Undergraduate Programme
   - □ Professional Education
   - □ Specialization Programme
   - □ Others:

4. **Total number of hours and distribution:**
   - x Theoretical Sessions: 225
   - □ Clinical/Other Placement:
   - □ Practice Seminars: 75
   - □ Lab Seminars:
   - □ Others:

* Total number of hours: 300

5. **Course/Module programme discipline:**
   - x Nursing

   - □ Medicine
   - □ Social work
   - □ Psychology
   - □ Physiotherapy
   - □ Occupational Therapy
   - □ Pharmacology
   - □ Nutrition
   - □ Others

6. **Course/Module programme´s topic/specialization (e.g. community nursing):**
   Interpersonal communication. Quality as the core value of the Health Systems. Management tools. Process management and Clinical pathways. Designing a Clinical pathway (design matrix/structure). Management and Administration specific Strategies. Discussion on sustainability strategies such as healthcare co-payment through Newspapers, Published Documents and so on. Healthcare Quality Indicators and Quality Assessment. Patient classification systems. Nursing workloads.

7. **Course/module organizing institution:** Faculty of Health Sciences. University of Alicante.
8. **Course/module settings:**
   - x Theoretical Education setting: university

   - x Practice Education setting: university

9. **Course/module edition number:** 3 (Since Bologna Plan)

10. **Course/module total number of students**: 200

11. **Course/module responsible professors/teachers:** 1

12. **Course/module professors´/teachers´ education background on HIS (specify the title):**
    - □ PhD/DNP Programme: Doctor in Public Health
    - □ Master Degree:
    - □ Postgraduate Programme:
    - □ Graduate Programme:
    - □ Undergraduate Programme:
    - □ Professional Education:
    - □ Specialization Programme:
    - □ Research experience on HIS:

- ◻ Others:

GENERAL INFORMATION ABOUT HIS STUDENTS/ALUMNI (TO BE FILLED BY STUDENTS/ALUMNI)

13. Please indicate your professional role:

- ◻ Healthcare assistant
- x Registered Nurse (RN)

- ◻ Specialized Nurse (including Nurse Practitioner)
- ◻ General Practitioner (GP)
- ◻ Specialized Medical Doctor (MD)
- ◻ Managerial/senior consultant
- ◻ Administrative
- ◻ Social worker
- ◻ Psychologist
- ◻ Physiotherapist
- ◻ Occupational Therapist
- ◻ Pharmacologist
- ◻ Nutritionist
- ◻ Others:

14. Please indicate your academic level:

- ◻ PhD/DNP :
- ◻ Master Degree:
- ◻ Postgraduate:
- ◻ Graduate:

x Undergraduate: Nursing Degree

- ◻ Specialization:
- ◻ Others:

15. Sex:

15.1 Man

x Woman

16. Age: 21

17. Previous HIS experience:

17.1 No

17.2 Yes:

- ◻ Experience in hours:
- ◻ Type of education programme:
- ◻ Name of the programme:

SPANISH FRONT PAGE. 2 :

GENERAL INFORMATION ABOUT HIS MODULE/COURSE (TO BE FILLED BY TUTORS/MENTORS)

***Please, specify the module/course title. Choose one of these two options:**

1. **Healthcare Improvement Science module/course (those using the SPECIFIC concept "Healthcare Improvement Science"):**

2. **Other modules/courses (those not using the SPECIFIC concept "Healthcare Improvement Science"):**

   Nursing

   2.1 If you have chosen this option, please tick the content/s included in that module/course (indirectly related to HIS):

   - ❏ Systems thinking and process mapping.
   - ❏ Models for improvement.
   - ❏ Measurement for improvement.
   - x Communication and managing change.

***Please, specify the module/course characteristics:**

3. **Programme education level:**
   - □ PhD/DNP Programme
   - □ Master Degree
   - □ Postgraduate Programme
   - □ Graduate Programme
   - x Undergraduate Programme
   - □ Professional Education
   - □ Specialization Programme
   - □ Others:

4. Total number of hours and distribution:
   - x Theoretical Sessions: 75
   - x Clinical/Other Placement: 150
   - x Practice Seminars: 75
   - □ Lab Seminars:
   - □ Others:

* Total number of hours: 300

5. **Course/Module programme discipline:**

x Nursing

- □ Medicine
- □ Social work
- □ Psychology
- □ Physiotherapy
- □ Occupational Therapy
- □ Pharmacology
- □ Nutrition
- □ Others

6. **Course/Module programme´s topic/specialization (e.g. community nursing):**
Base the interventions of Health Science professionals on scientific evidence and the available resources. Apply the necessary methods and procedures in your field to identify health problems. Identify and understand the experience of suffering from a chronic process (or illness) and being dependent. Show oral and written communication skills.

7. **Course/module organizing institution**: Faculty of Health Sciences. University of Alicante.
8. **Course/module settings**:

x Theoretical Education setting: University

x Practice Education setting: Hospital

9. **Course/module edition number**: 2

10. **Course/module total number of students**: 200

11. **Course/module responsible professors/teachers**: 1

12. **Course/module professors´/teachers´ education background on HIS (specify the title):**
- □ PhD/DNP Programme: "Enfermería: Práctica y Educación"
- □ Master Degree: Master in Nursing Sciences
- □ Postgraduate Programme:
- □ Graduate Programme:
- □ Undergraduate Programme:
- □ Professional Education:
- □ Specialization Programme:
- □ Research experience on HIS: health outcomes research, evidence based practice in palliative care and oncology

- ◻ Others:

GENERAL INFORMATION ABOUT HIS STUDENTS/ALUMNI (TO BE FILLED BY STUDENTS/ALUMNI)

13. Please indicate your professional role:

- ◻ Healthcare assistant
- x Registered Nurse (RN)
- ◻ Specialized Nurse (including Nurse Practitioner)
- ◻ General Practitioner (GP)
- ◻ Specialized Medical Doctor (MD)
- ◻ Managerial/senior consultant
- ◻ Administrative
- ◻ Social worker
- ◻ Psychologist
- ◻ Physiotherapist
- ◻ Occupational Therapist
- ◻ Pharmacologist
- ◻ Nutritionist
- ◻ Others:

14. Please indicate your academic level:

- ◻ PhD/DNP :
- ◻ Master Degree:
- ◻ Postgraduate:
- ◻ Graduate:
- x Undergraduate: Nursing Degree + Biology Degree
- ◻ Specialization:
- ◻ Others:

15. Sex:

15.1 Man
x Woman

16. Age: 40

17. Previous HIS experience:

17.1 No
17.2 Yes x

- ◻ Experience in hours:
- ◻ Type of education programme:
- ◻ Name of the programme:

ROMANIAN FRONT PAGE. 1 :

GENERAL INFORMATION ABOUT HIS MODULE/COURSE (TO BE FILLED BY TUTORS/MENTORS)

***Please, specify the module/course title. Choose one of these two options:**

1. **Healthcare Improvement Science module/course (those using the SPECIFIC concept "Healthcare Improvement Science"):**

2. **Other modules/courses (those not using the SPECIFIC concept "Healthcare Improvement Science"):**

   Public Health and Management

   2.1 If you have chosen this option, please tick the content/s included in that module/course (indirectly related to HIS):

   ❏ Systems thinking and process mapping.
   X Models for improvement.
   X Measurement for improvement.
   x Communication and managing change.

***Please, specify the module/course characteristics:**

3. **Programme education level:**
   - □ PhD/DNP Programme
   - □ Master Degree
   - □ Postgraduate Programme
   - □ Graduate Programme
   - x Undergraduate Programme
   - □ Professional Education
   - □ Specialization Programme
   - □ Others:

4. Total number of hours and distribution:
   - x Theoretical Sessions: 21
   - □ Clinical/Other Placement:
   - x Practice Seminars: 21
   - □ Lab Seminars:
   - □ Others:

   * Total number of hours: 42

5. **Course/Module programme discipline:**
   - ◻ Nursing

   X Medicine
   - ◻ Social work
   - ◻ Psychology
   - ◻ Physiotherapy
   - ◻ Occupational Therapy
   - ◻ Pharmacology
   - ◻ Nutrition
   - ◻ Others

6. **Course/Module programme´s topic/specialization (e.g. community nursing):**

   Health management
7. **Course/module organizing institution**: Gr T Popa University of Medicine and Pharmacy
8. **Course/module settings**:
   - ◻ Theoretical Education setting: University

   - ◻ Practice Education setting: Hospital

9. **Course/module edition number**:

10. **Course/module total number of students**:

11. **Course/module responsible professors/teachers**:

12. **Course/module professors´/teachers´ education background on HIS (specify the title):**
    - ◻ PhD/DNP Programme:
    - ◻ Master Degree:
    - ◻ Postgraduate Programme:
    - ◻ Graduate Programme:
    - ◻ Undergraduate Programme:
    - ◻ Professional Education:
    - ◻ Specialization Programme:
    - ◻ Research experience on HIS:
    - ◻ Others:

GENERAL INFORMATION ABOUT HIS STUDENTS/ALUMNI (TO BE FILLED BY STUDENTS/ALUMNI)

13. **Please indicate your professional role:**
    - ◻ Healthcare assistant
    - ◻ Registered Nurse (RN)
    - ◻ Specialized Nurse (including Nurse Practitioner)

- □ General Practitioner (GP)
- □ Specialized Medical Doctor (MD)
- □ Managerial/senior consultant
- □ Administrative
- □ Social worker
- □ Psychologist
- □ Physiotherapist
- □ Occupational Therapist
- □ Pharmacologist
- □ Nutritionist
- □ Others: Medicine Student

14. Please indicate your academic level:

- □ PhD/DNP :
- □ Master Degree:
- □ Postgraduate:
- □ Graduate:

x Undergraduate:

- □ Specialization:
- □ Others:

15. Sex:

X Man

15.1 Woman

16. Age: 25

17. Previous HIS experience:

17.1 No

17.2 Yes x

- □ Experience in hours:
- □ Type of education programme:

ROMANIAN FRONT PAGE. 2 :

GENERAL INFORMATION ABOUT HIS MODULE/COURSE (TO BE FILLED BY TUTORS/MENTORS)

***Please, specify the module/course title. Choose one of these two options:**

1. **Healthcare Improvement Science module/course (those using the SPECIFIC concept "Healthcare Improvement Science"):**

2. **Other modules/courses (those not using the SPECIFIC concept "Healthcare Improvement Science"):**

Bioethics

2.1 If you have chosen this option, please tick the content/s included in that module/course (indirectly related to HIS):

- ❑ Systems thinking and process mapping.
- ❑ Models for improvement.
- ❑ Measurement for improvement.
- x Communication and managing change.

*Please, specify the module/course characteristics:

3. Programme education level:

- ◻ PhD/DNP Programme
- ◻ Master Degree
- ◻ Postgraduate Programme
- ◻ Graduate Programme
- x Undergraduate Programme
- ◻ Professional Education
- ◻ Specialization Programme
- ◻ Others:

4. Total number of hours and distribution:

- x Theoretical Sessions: 14
- ◻ Clinical/Other Placement:
- x Practice Seminars: 14
- ◻ Lab Seminars:
- ◻ Others:

* Total number of hours: 28

5. Course/Module programme discipline:

- ◻ Nursing
- X Medicine
- ◻ Social work
- ◻ Psychology
- ◻ Physiotherapy
- ◻ Occupational Therapy
- ◻ Pharmacology
- ◻ Nutrition
- ◻ Others

6. **Course/Module programme´s topic/specialization (e.g. community nursing):**

   Deontology and Bioethics
7. **Course/module organizing institution**: Gr T Popa University of Medicine and Pharmacy
8. **Course/module settings**:
   - □ Theoretical Education setting: University
   - □ Practice Education setting: Hospital
9. **Course/module edition number**:
10. **Course/module total number of students**:
11. **Course/module responsible professors/teachers**:
12. **Course/module professors´/teachers´ education background on HIS (specify the title):**
   - □ PhD/DNP Programme:
   - □ Master Degree:
   - □ Postgraduate Programme:
   - □ Graduate Programme:
   - □ Undergraduate Programme:
   - □ Professional Education:
   - □ Specialization Programme:
   - □ Research experience on HIS:
   - □ Others:

GENERAL INFORMATION ABOUT HIS STUDENTS/ALUMNI (TO BE FILLED BY STUDENTS/ALUMNI)

13. **Please indicate your professional role:**
   - □ Healthcare assistant
   - □ Registered Nurse (RN)
   - □ Specialized Nurse (including Nurse Practitioner)
   - □ General Practitioner (GP)
   - □ Specialized Medical Doctor (MD)
   - □ Managerial/senior consultant
   - □ Administrative
   - □ Social worker
   - □ Psychologist
   - □ Physiotherapist
   - □ Occupational Therapist
   - □ Pharmacologist
   - □ Nutritionist
   - □ Others: Medicine Student

14. Please indicate your academic level:

- ◻ PhD/DNP :
- ◻ Master Degree:
- ◻ Postgraduate:
- ◻ Graduate:

x Undergraduate:

- ◻ Specialization:
- ◻ Others:

15. Sex:

15.1 Man

X Woman

16. Age: 23

17. Previous HIS experience:

17.1 No

17.2 Yes x

- ◻ Experience in hours:
- ◻ Type of education programme:

ROMANIAN FRONT PAGE. 3 :

GENERAL INFORMATION ABOUT HIS MODULE/COURSE (TO BE FILLED BY TUTORS/MENTORS)

***Please, specify the module/course title. Choose one of these two options:**

1. **Healthcare Improvement Science module/course (those using the SPECIFIC concept "Healthcare Improvement Science"):**

2. **Other modules/courses (those not using the SPECIFIC concept "Healthcare Improvement Science"):**

Family Medicine

2.1 If you have chosen this option, please tick the content/s included in that module/course (indirectly related to HIS):

- ❏ Systems thinking and process mapping.
- ❏ Models for improvement.
- ❏ Measurement for improvement.
- x Communication and managing change.

*Please, specify the module/course characteristics:

3. **Programme education level:**
   - □ PhD/DNP Programme
   - □ Master Degree
   - □ Postgraduate Programme
   - □ Graduate Programme
   - □ Undergraduate Programme
   - □ Professional Education
   - X Specialization Programme
   - □ Others:

4. Total number of hours and distribution:
   - x Theoretical Sessions:
   - □ Clinical/Other Placement:
   - x Practice Seminars:
   - □ Lab Seminars:
   - □ Others:

   * Total number of hours: 200 hours

5. **Course/Module programme discipline:**
   - □ Nursing
   - X Medicine
   - □ Social work
   - □ Psychology
   - □ Physiotherapy
   - □ Occupational Therapy
   - □ Pharmacology
   - □ Nutrition
   - □ Others

6. **Course/Module programme´s topic/specialization (e.g. community nursing):**

   Family Medicine

7. **Course/module organizing institution**: Gr T Popa University of Medicine and Pharmacy
8. **Course/module settings**:
   - □ Theoretical Education setting: Iasi University Hospitals
   - □ Practice Education setting: Iasi University Hospitals
9. **Course/module edition number**:

10. **Course/module total number of students**:

11. **Course/module responsible professors/teachers**:

12. **Course/module professors´/teachers´ education background on HIS (specify the title):**
    - ◻ PhD/DNP Programme:
    - ◻ Master Degree:
    - ◻ Postgraduate Programme:
    - ◻ Graduate Programme:
    - ◻ Undergraduate Programme:
    - ◻ Professional Education:
    - ◻ Specialization Programme:
    - ◻ Research experience on HIS:
    - ◻ Others:

GENERAL INFORMATION ABOUT HIS STUDENTS/ALUMNI (TO BE FILLED BY STUDENTS/ALUMNI)

13. **Please indicate your professional role:**
    - ◻ Healthcare assistant
    - ◻ Registered Nurse (RN)
    - ◻ Specialized Nurse (including Nurse Practitioner)
    - X General Practitioner (GP)
    - ◻ Specialized Medical Doctor (MD)
    - ◻ Managerial/senior consultant
    - ◻ Administrative
    - ◻ Social worker
    - ◻ Psychologist
    - ◻ Physiotherapist
    - ◻ Occupational Therapist
    - ◻ Pharmacologist
    - ◻ Nutritionist
    - ◻ Others: Medicine Student

14. **Please indicate your academic level:**
    - ◻ PhD/DNP :
    - ◻ Master Degree:
    - ◻ Postgraduate:
    - X Graduate:
    - ◻ Undergraduate:
    - ◻ Specialization:
    - ◻ Others:

15. Sex:

15.1 Man

X Woman

16. Age: 28

17. Previous HIS experience:

17.1 No

17.2 Yes x

- Experience in hours:
- Type of education programme:

PILOTING

HIS Learning Evaluation Framework

Level 1. Reaction. STUDENT

Reaction evaluation is how the participants felt, and their personal reactions to the course.

Please tick the one that most reflects your reaction.

1. Did you like the objectives planned for this course?

Very much	Somewhat	Undecided<br>Neutral	Not much<br>Not really	Not at all

Please describe it:

1.1. Would you add other objectives/contents to the course?

☐ YES

☐ NO

Please specify:

2. When you got the information about the course, you thought that the time planned was appropriate:

Very much — Somewhat — Undecided / Neutral — Not much / Not really — Not at all

3. Are there any aspects of the course you think will be particularly useful?

4. At first impression, did you like the course?

Very much — Somewhat — Undecided / Neutral — Not much / Not really — Not at all

Please specify:

5. In the first impression, did the course meet your expectations? Why?

6. Did you think the course would be useful for your professional future?

Very much — Somewhat — Undecided / Neutral — Not much / Not really — Not at all

6.1. What were you hoping to learn from this course?

- ☐ Improved understanding of ______________________________
- ☐ Updated knowledge of ______________________________
- ☐ Learnt how to apply techniques relating to ______________________________
- ☐ Changed how I think about ______________________________
- ☐ Help me relate better to ______________________________
- ☐ Other: ______________________________

Level 2. Learning. STUDENT

Learning evaluation is the measurement of the increase in knowledge or intellectual capability from before to after the learning experience. How knowledge or capability increases as a result of participating in the course.

To be fulfilled by students.

1. Do you think you have reached the objectives proposed?

- ☐ YES
- ☐ NO

Please specify:

2. Has the knowledge been taught clearly?

Very much	Somewhat	Undecided Neutral	Not much Not really	Not at all
○	○	○	○	○

2.1. Which method helped you learn most? Is there another methodology you could prefer?
Specify your reasons:

2.2. How did the materials provided by your educator improve your learning?
Specify your reasons:

3. To which extent did participation improve your learning?

Very much | Somewhat | Undecided Neutral | Not much Not really | Not at all

3.1. Were there enough opportunities for students to participate?

- ◻ YES
- ◻ NO

Please specify:

4. Was it easy to engage in the course?

Very much | Somewhat | Undecided Neutral | Not much Not really | Not at all

4.1. What helped you to engage in learning?

5. How was the learning experience?

6. Are you satisfied about how much you have learnt?

Very much | Somewhat | Undecided Neutral | Not much Not really | Not at all

7. Are you satisfied about what you have learnt?

Very much	Somewhat	Undecided Neutral	Not much Not really	Not at all

8. What have you learnt from this course?

- ☐ Improved understanding of ______________________________
- ☐ Update knowledge of ______________________________
- ☐ Learn how to apply techniques relating to ______________________________
- ☐ Change how I think about ______________________________
- ☐ Help me relate better to ______________________________
- ☐ Other: ______________________________

9. What has been modified most after the learning? (order the items from 1 to 4 considering 1 as the most important)

- ☐ Skills
- ☐ Knowledge
- ☐ Attitudes
- ☐ Others

9.1. Could you please describe how those skills were modified?

9.2. Could you please describe how knowledge was modified?

9.3. Could you please describe how those attitudes were modified?

____

10. What other contents would you add to the course?

11. What contents would you eliminate from the course?

12. In general, do you consider the new knowledge useful?

- ☐ YES
- ☐ NO

Please specify:

13. What do you plan to do with your knew knowledge? Could you apply it?

14. In general, I enjoyed the course:

Very much	Somewhat	Undecided<br>Neutral	Not much<br>Not really	Not at all

15. The most remarkable educator characteristics: (order from 1 to 5)

- □Motivation
- □Teaching ability
- □Knowledge of the topic
- □Field experience
- □Closeness, empathy

16. The educator improved my learning process:

Very much Somewhat Undecided Neutral Not much Not really Not at all

17. The time dedicated was adequate:

Very much Somewhat Undecided Neutral Not much Not really Not at all

Level 3. Behavior/ Training transfer. STUDENT

Behavior evaluation is the extent to which the trainees applied the learning and changed their behavior, and this can be immediately and several months after the course**, depending on the situation.** This section is about finding out **what elements** of the course and to **what extent** they applied the content in the workplace.

1. After the course, are you able to develop HIS actions in other contexts?
   - ☐ YES
   - ☐ NO

   Please specify:

2. In which degree was the course content necessary in the workplace?

Very much Somewhat Undecided Neutral Not much Not really Not at all

2.1. Have you been able to transfer in your workplace the knowledge received during the course?
   - ☐ YES
   - ☐ NO

Please specify:

2.2. Were the course objectives adapted to your workplace?

☐ YES

☐ NO

Please specify:

2.3. Were the course competences adapted to your workplace?

☐ YES

☐ NO

Please specify:

2.4. Were the course activities adapted to your workplace?

☐ YES

☐ NO

Please specify:

3. In which degree do you change in your behavior, knowledge, skill level?

Very much	Somewhat	Undecided Neutral	Not much Not really	Not at all

Please, give an example

4. In which degree do you apply the knowledge/skills acquired through the course in

your workplace?

Very much | Somewhat | Undecided Neutral | Not much Not really | Not at all

Please, give an example of the most relevant skills/knowledge/attitudes you have applied:

5. In which degree was difficult to apply what you have learnt to the workplace?

Very much | Somewhat | Undecided Neutral | Not much Not really | Not at all

Please, describe your reasons:

5.1. What can educators do to support students overcome barriers to application?

5.2. Can you describe how your workplace context limits the potential for HIS? And how could those barriers be replaced?

6. What are you doing differently now as a result of being on the course?

HIS Learning Evaluation Framework

Level 4. Results. STUDENTS

In this level we want to describe how the practice has got to outcomes and impact of transferring the learning, to what extent results have been affected by the course**. Results evaluation** is the **effect on the context** resulting from the improved performance of the trainee.

1. In which degree has HIS course affected daily practice in the workplace?

Very much	Somewhat	Undecided<br>Neutral	Not much<br>Not really	Not at all

1.1. What kind of changes have you perceived?
Please, classify those changes in positive and negative ones:

1.2. Can you describe the evidence of those changes (Indicators, percentages, timescales, ratings, achievement of standards and accreditations, and other quantifiable aspects of organizational performance)? Is it impact or non-measurable outcomes instead of measurable results?

2. In which degree have you experienced changes in behavior/knowledge/skills in other colleagues at the workplace?

Very much	Somewhat	Undecided<br>Neutral	Not much<br>Not really	Not at all

Please specify:

3. In which degree do you think your colleagues have applied the methods learnt?

Very much	Somewhat	Undecided<br>Neutral	Not much<br>Not really	Not at all

Please specify:

4. In which degree has HIS learning practice affected the use of resources in the workplace?

Very much — Somewhat — Undecided Neutral — Not much Not really — Not at all

Please specify:

5. In which degree has HIS course affected the workplace quality perceived?

Very much — Somewhat — Undecided Neutral — Not much Not really — Not at all

Please specify:

6. In your opinion, what kind of skills/attitudes/knowledge learnt has changed results mostly in the workplace?

7. In which degree do you think the change in your behavior has affected the organization?

Strongly Agree	Agree	Neutral	Disagree	Strongly Disagree

Please, describe your reasons and examples:

8. What benefits have you perceived as a consequence of applying HIS course in the workplace?

Level 5. Return on investment. STUDENT

In this level we want to evaluate the return of investment after the process, understood as an ongoing quality improvement process.

1. Have you been doing something (projects, courses, conferences…) related to HIS since you have finished until now ?
   - ☐ YES
   - ☐ NO

   Please specify:

2. Have you collected results that could justify the efficiency of HIS actions developed after the course?
   - ☐ YES
   - ☐ NO

   Please specify:

3. To what extent have you disseminated or exploited (through publications, for instance) HIS knowledge?

Very much | Somewhat | Undecided Neutral | Not much Not really | Not at all

Please specify:

4. To what extent have you taught others on HIS knowledge/techniques/methods?

Very much | Somewhat | Undecided Neutral | Not much Not really | Not at all

Please specify:

5. To what extent have you promoted the use or interest on HIS education in your context?

Very much | Somewhat | Undecided Neutral | Not much Not really | Not at all

Please specify:

5.1. How do you plan in the future to keep on developing the HIS skills/attitudes/knowledge you have learned?

HIS Learning Evaluation Framework

Level 1. Reaction. EDUCATOR

Reaction evaluation is how the educators felt, and their personal reactions to the course.

Please tick the one that most reflects your reaction

1. Did you think the students liked the objectives planned for this course?

Very much — Somewhat — Undecided Neutral — Not much Not really — Not at all

1.1. What did you think about the objectives you have planned for this course?

2. How would you describe the students first reaction?

3. Did you think the time planned for the course was appropriate?

Very much — Somewhat — Undecided Neutral — Not much Not really — Not at all

4. Did you think the course will be useful for the students professional future?

Very much — Somewhat — Undecided Neutral — Not much Not really — Not at all

HIS Learning Evaluation Framework

Level 2. Learning. EDUCATOR

Learning evaluation is the measurement of the increase in knowledge or intellectual capability from before to after the learning experience. How did knowledge or capability increase as a result of participating in the course.

To be fulfilled by EDUCATOR.

1. Do you think the students have reached the objectives proposed?

   ☐ YES
   ☐ NO

Please specify:

2. Do you think the students experienced, through the course, what was supposed to be experienced?

Very much	Somewhat	Undecided<br>Neutral	Not much<br>Not really	Not at all

2.1. Which method helped them learn most?

2.2. How did the materials provide improve the students' learning?

3. To which extent did participation improve the students' learning?

Very much	Somewhat	Undecided<br>Neutral	Not much<br>Not really	Not at all

4. Did you receive feedback about what they have learnt?

   ☐ YES
   ☐ NO

Please specify:

4. Are you satisfied about how much they have learnt?

Very much	Somewhat	Undecided Neutral	Not much Not really	Not at all

5. The time dedicated was adequate:

Very much	Somewhat	Undecided Neutral	Not much Not really	Not at all

6. What other content would you add to the course?

7. What content would you eliminate from the course?

HIS Learning Evaluation Framework

Level 3. Behavior/ Training transfer. EDUCATOR

Behavior evaluation is the extent to which the trainees applied the learning and changed their behavior, and this can be immediately and several months after the course**, depending on the situation.** This section is about finding out **what elements** of the course and to **what extent** they applied the content in the workplace.

1. In which degree do the students behavior/knowledge/skills level change?

Very much | Somewhat | Undecided Neutral | Not much Not really | Not at all

Please, give an example

1.1. What can educators do to support students overcome barriers to context application?

2. In which degree do you think the students have applied the methods learnt?

Very much | Somewhat | Undecided Neutral | Not much Not really | Not at all

3. Did you received feedback about what they have transferred?

- ☐ YES
- ☐ NO

Please specify:

HIS Learning Evaluation Framework

Level 4. Results. EDUCATOR

In this level we want to describe how the practice has got to outcomes and impact of transferring the learning, to what extent results have been affected by the course**. Results evaluation** is the **effect on the context** resulting from the improved performance of the trainee.

1. What kind of outcomes have you perceived?
Please, classify those changes in positive and negative ones:

1.3. Can you describe the evidence of those outcomes (Indicators, percentages, timescales, ratings, achievement of standards and accreditations, and other quantifiable aspects of organizational performance)? Is it impact or non-measurable outcomes instead of measurable results?

1.4. In your opinion, what kind of learnt (skills/attitudes/knowledge) have changed results mostly?

2. What are you doing differently now as a result of being their educator?

HIS Learning Evaluation Framework

Level 5. Return on investment. MANAGER

In this level we want to evaluate the return of investment after the process, understood as an ongoing quality improvement process.

1. Have you collected results that could justify the efficiency of HIS actions developed after the course?
   - ☐ YES
   - ☐ NO

   Please specify:

2. To what extent have you disseminated or exploited (through publications, for instance) HIS knowledge?

Very much	Somewhat	Undecided<br>Neutral	Not much<br>Not really	Not at all

Please specify:

3. To what extent have you taught others on HIS knowledge/techniques/methods?

Very much	Somewhat	Undecided<br>Neutral	Not much<br>Not really	Not at all

Please specify:

4. To what extent have you promoted the use or interest on HIS education in your context?

Very much	Somewhat	Undecided<br>Neutral	Not much<br>Not really	Not at all

Please specify:

5. After the course, have you been doing something else than this course (projects, courses, conferences…) related to HIS?

☐ YES

☐ NO

Please specify:

HIS Learning Evaluation Framework

Level 3. Behavior/ Training transfer. MANAGER

Behavior evaluation is the extent to which the trainees applied the learning and changed their behavior, and this can be immediately and several months after the course**, depending on the situation.** This section is about finding out **what elements** of the course and to **what extent** they applied the content in the workplace.

1. In which degree do the students apply the knowledge/skills acquired through the course in the workplace?

Very much	Somewhat	Undecided<br>Neutral	Not much<br>Not really	Not at all

Please, give an example

1.1. What, in your opinion, are the most relevant skills/knowledge/attitudes the students have applied to the workplace?

2. In which degree was difficult to apply what the students have learnt to the workplace?

Very much	Somewhat	Undecided<br>Neutral	Not much<br>Not really	Not at<br>all

Please, describe your reasons:

2.1. Can you describe how the workplace context limits the potential for HIS? And how could those barriers be replaced?

HIS Learning Evaluation Framework

Level 4. Results. MANAGER

In this level we want to describe how the practice has got to outcomes and impact of transferring the learning, to what extent results have been affected by the course**. Results evaluation** is the **effect on the context** resulting from the improved performance of the trainee.

1. In which degree has HIS course affected daily practice in the workplace?

Very much	Somewhat	Undecided<br>Neutral	Not much<br>Not really	Not at all

1.5. What kind of changes have you perceived?
Please, classify those changes in positive and negative ones:

1.6. Can you describe the evidence of those changes (Indicators, percentages, timescales, ratings, achievement of standards and accreditations, and other quantifiable aspects of organizational performance)? Is it impact or non-measurable outcomes instead of measurable results?

2. In which degree has HIS learning affected the use of resources in the workplace?

Very much	Somewhat	Undecided<br>Neutral	Not much<br>Not really	Not at all

Please specify:

3. In which degree has the HIS course in practice affected the workplace quality perceived?

Very much — Somewhat — Undecided Neutral — Not much Not really — Not at all

Please specify:

3.1. In your opinion, what kind of learnt (skills/attitudes/knowledge) have changed results mostly in the workplace?

4. In which degree do you think the students change in their behavior has affected the organization?

Strongly Agree — Agree — Neutral — Disagree — Strongly Disagree

Please, describe your reasons and examples:

4.1. What benefits have you perceived as a consequence of applying HIS course in the workplace?

5. In which degree have you experienced changes in behavior/knowledge/skills in other colleagues at the workplace?

Very much — Somewhat — Undecided Neutral — Not much Not really — Not at all

Please specify:

6. In which degree do you think their colleagues have applied the methods learnt?

Very much | Somewhat | Undecided Neutral | Not much Not really | Not at all

Please specify:

7. What are you doing differently now as a result of being part of the learning process as a manager?

HIS Learning Evaluation Framework

Level 5. Return on investment. MANAGER

In this level we want to evaluate the return of investment after the process, understood as an ongoing quality improvement process.

1. Have you collected results that could justify the efficiency of HIS actions developed after the course?
   - ☐ YES
   - ☐ NO

   Please specify:

2. To what extent have you disseminated or exploited (through publications, for instance) HIS knowledge?

Very much | Somewhat | Undecided Neutral | Not much Not really | Not at all

Please specify:

3. To what extent have you taught others on HIS knowledge/techniques/methods?

Very much | Somewhat | Undecided Neutral | Not much Not really | Not at all

Please specify:

4. To what extent have you promoted the use or interest on HIS education in your context?

Very much | Somewhat | Undecided Neutral | Not much Not really | Not at all

Please specify:

5. After participating as a manager, have you been doing something else (projects, courses, conferences…) related to HIS?
   - ☐ YES
   - ☐ NO

Please specify:

HIS Learning Evaluation Framework

Level 3. Behavior/ Training transfer. MENTOR

Behavior evaluation is the extent to which the trainees applied the learning and changed their behavior, and this can be immediately and several months after the course**, depending on the situation.** This section is about finding out **what elements** of the course and to **what extent** they applied the content in the workplace.

1. In which degree was the course content necessary in the workplace?

Very much	Somewhat	Undecided<br>Neutral	Not much<br>Not really	Not at all

1.1. Have the students been able to transfer in the workplace the knowledge received during the course?

☐ YES

☐ NO

Please specify:

1.2. Were the course objectives adapted to the workplace?

☐ YES

☐ NO

Please specify:

1.3. Were the course competences adapted to the workplace?

☐ YES

☐ NO

Please specify:

1.4. Were the course activities adapted to the workplace?

- ☐ YES
- ☐ NO

Please specify:

2. In which degree do the students apply the knowledge/skills acquired through the course in the workplace?

Very much	Somewhat	Undecided<br>Neutral	Not much<br>Not really	Not at all

Please, give examples

2.1. What, in your opinion, are the most relevant skills/knowledge/attitudes they have applied to the workplace?

3. In which degree was difficult to apply what the students have learnt to the workplace?

Very much	Somewhat	Undecided<br>Neutral	Not much<br>Not really	Not at all

Please, describe your reasons:

3.1. What can the educators do to support students overcome barriers to application?

3.2. Can you describe how the workplace context limits the potential for HIS? And how could those barriers be replaced?

HIS Learning Evaluation Framework

Level 4. Results. MENTOR

In this level we want to describe how the practice has got to outcomes and impact of transferring the learning, to what extent results have been affected by the course**. Results evaluation** is the **effect on the context** resulting from the improved performance of the trainee.

1. In which degree has HIS course affected daily practice in the workplace?

Very much	Somewhat	Undecided<br>Neutral	Not much<br>Not really	Not at all

1.1. What kind of changes have you perceived?
Please, classify those changes in positive and negative ones:

1.2. Can you describe the evidence of those changes (Indicators, percentages, timescales, ratings, achievement of standards and accreditations, and other quantifiable aspects of organizational performance)? Is it impact or non-measurable outcomes instead of measurable results?

2. In which degree has HIS learning practice affected the use of resources in the workplace?

Very much — Somewhat — Undecided Neutral — Not much Not really — Not at all

Please specify:

3. In which degree has HIS course in practice affected the workplace quality perceived?

Very much — Somewhat — Undecided Neutral — Not much Not really — Not at all

Please specify:

3.1. In your opinion, what kind of learnt (skills/attitudes/knowledge) have changed results mostly in the workplace?

4. In which degree do you think the change in their behavior has affected the organization?

Strongly Agree | Agree | Neutral | Disagree | Strongly Disagree

Please, describe your reasons and examples:

4.1. What benefits have you perceived as a consequence of applying HIS course in the workplace?

5. In which degree have you experienced changes in behavior/knowledge/skills in other colleagues at the workplace?

Very much | Somewhat | Undecided Neutral | Not much Not really | Not at all

Please specify:

6. In which degree do you think their colleagues have applied the methods learnt?

Very much | Somewhat | Undecided Neutral | Not much Not really | Not at all

Please specify:

7. What are you doing differently now as a result of being their mentor?

HIS Learning Evaluation Framework

Level 5. Return on investment. MENTOR

In this level we want to evaluate the return of investment after the process, understood as an ongoing quality improvement process.

1. Have you collected results that could justify the efficiency of HIS actions developed after the course?
   - ☐ YES
   - ☐ NO

   Please specify:

2. To what extent have you disseminated or exploited (through publications, for instance) HIS knowledge?

   Very much — Somewhat — Undecided Neutral — Not much Not really — Not at all

   Please specify:

3. To what extent have you taught others on HIS knowledge/techniques/methods?

   Very much — Somewhat — Undecided Neutral — Not much Not really — Not at all

   Please specify:

4. To what extent have you promoted the use or interest on HIS education in your context?

Very much	Somewhat	Undecided<br>Neutral	Not much<br>Not really	Not at all

Please specify:

5. After the course, have you been doing something else than this course (projects, courses, conferences…) related to HIS?
   - ☐ YES
   - ☐ NO

   Please specify:

DISCUSSION ABOUT DEVELOPING AND PILOTING THE HEALTHCARE IMPROVEMENT SCIENCE LEARNING EVALUATION FRAMEWORK:

These are the key conclusions that came up from both the developing and piloting process during the whole WP10 duration:

- As all partners agreed through the whole developing proccess, the questionnaire was **considerably long** and the questions per level should had been separated or reduced unless an online questionnaire would be developed or at least different shorter questionnaires could be extracted. When the piloting process began, the questionnaire had already been classified in different levels and focused on different types of participants so that each partner team could select the specific questionnaire for the specific sample they were piloting with. In all cases of the piloting, a front page containing a MDS was compulsory completed about the items or variables describing the sort of Education Module or Course. Finally, in the last Skype Meeting discussion held on the 24th of July 2015, the Spanish team suggested to create a Google Form template so that it could be easier for participants accross the different countries to complete the questionnaire even through their own cell phone. That way, the ISTEW team would receive the information feedback directly and would be provided with a quantitative and qualitative analysis format. That option would also provide the ISTEW team with a tool that would monitor participants even in the future, at level 5.

- Regarding the specific students questionnaire at different levels, some partners reported that after the first ten minutes the students didn't answer with the same accuracy as they did in the beggining. After those comments, the questionnaire was divided in different levels for different time moments, and the style of writting was reviewed to remain easy language.

- In the developing process, partners agreed that some **subquestions** (secondary questions) **should be lef out** in order to remain clear for participants to understand. All partners also agreed that there was no need to go deeper into subquestions. Consequently, different questions that might lead to similar answers were also deleted.

- The **complexity** of the questionnaire has been discussed along the piloting process. Not all students were capable to answer in the expected way. **Post-graduate samples** seemed to have a previous critical thinking training to answer to the questionnare and also to the topic understanding. One limitation that came up in this point is the background of the sample used. Future piloting should be implemented with other samples with distinct education backgrounds and at different levels. Another limitation is related to the fact that most of the samples came from areas of Nursing (in 6 partner countries), Medicine (in 1 partner country, Romania) and Psychology (in 1 partner country, Poland). More testing with other disciplines related to health and social care should be implemented.

- The **sample selection** was one of the main limitations due to the **language barrier** as in the 5 countries where English was not the mother language, partner teams had to select those participants that could at least read and write in English. That point was connected to the motivation and also to the **previous knowledge about HIS**. Romanian Team suggested that complete samples should be used, not only those considered as English speakers. Spanish Students suggested the importance of including an **introduction** for a better understanding of the piloting aim and also about HIS concept. In the future and after validating the questionnaire, a transcultural adaptation of the tool should be implemented.

- All colleagues agreed that a **grammar review** was required/necessary. It is important to remark that before starting the piloting, several grammar reviews were developed in order to produce a questionnaire that could be easily and clearly understood by participants at the different levels, especially for students.

- The **format** could be improved through an online platform or even the box and letter sizes. A consensus about the **questions style** (open questions, multi option questions or closed questions) was not achieved, as each partner and even each participant had a different opinion, in different countries. It is important to remark that in the ongoing of creating the tool, all

partners agreed to finally have open questions and closed questions (with a likert scale). In fact, in the current questionnaire the same thing is asked twice along the questionnaire with the two types of questions to validate the content of the answers.

- All the piloting results concluded with a **good overview** of what was the objective of the questionnaire and which were the main topics the questions were asking about. In general, all participants were able to understand all the questions and also to capture the differences between levels.
- **Time** has been considered as one of the main limitations in the piloting process. For example, level 5 should be piloted once HIS Education had finished and consequently the students had enough time to carry out some exploitation activities in the workplace to evalute the return on the HIS education investment. On the other hand, some partners refered to the problem of Time also in the task of filling the questionnaire, however in the last Skype Meeting held on the 24th of July 2015, partners concluded that the problem of time was directly linked with the fact that the piloting was developed in English in all the countries, while in 5 out of the 7 countries the mother language was another one and obviously the participants had to make the effort to read and understand in another language. The comment about Time is also associated to another important fact which is that Healthcare Improvement Science (HIS) was something new for most of the partner countries. Concretely, only Scotland and UK had specific literature published about Healthcare Improvement Science while for the rest of countries it was something new and participants had a doble effort in understanding English and understanding about the concepts related to HIS.

LIMITATIONS ASSOCIATED TO THE DEVELOPMENT OF THE HEALTHCARE IMPROVEMENT SCIENCE EVALUATION FRAMEWORK:

After the whole process, it is not possible to evaluate the Healthcare Improvement Science modules that the ISTEW partner teams are still developing rather they are developing a framework through a process of consensus and piloting the framework contents through a content validation in each of the 7 partner countries. This situation will allow partner countries to evaluate the modules as they are implemented in their own countries. It is important to remark that the Evaluation Framework tool has been developed in English and 5 of the 7 partner teams have another mother language. On the other hand, until now, the cases of Education Modules used were not Healthcare Improvement Science Modules or Courses. They were all

modules or courses that contained items related to HIS but were not real HIS modules.

FUTURE METHODOLOGICAL ONGOING AND IMPLICATIONS:

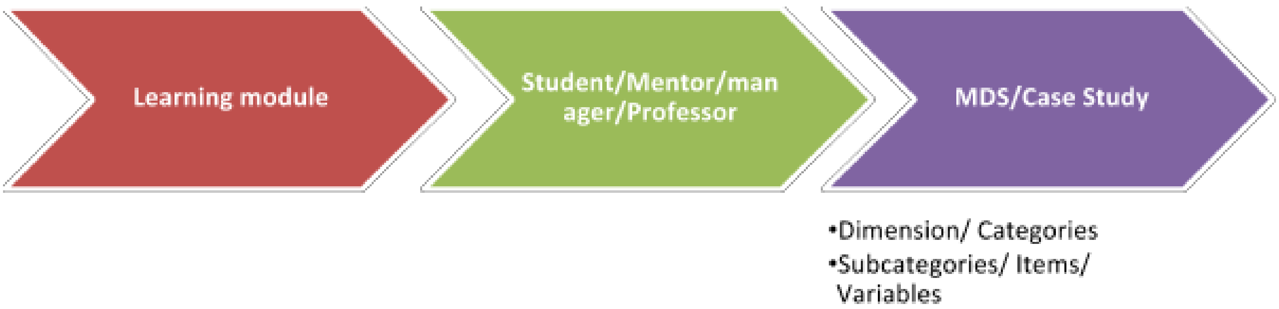

After the development of the 4 Healthcare Improvement Science Modules developed within ISTEW Project and as part of the Project's outcomes of WP8 and that will be future tested in the different partner countries, the Healthcare Improvement Science Evaluation Framework developed until now and presented through WP10 should then be piloted on the base of a real Healthcare Improvement Science Training, with real cases corresponding to real HIS modules. That long-term evaluation (on the scope of HIS modules) will enable an appropiate evaluation and also will justify the final European HIS framework. In addition, and out of the ISTEW research objectives, the Spanish partner team aware of the prospective scope of this evaluation and also to improve the data collection process, suggested to develop a google form tool (attached at the end) for each specific questionnaire designed for each level and each type of participant so that ISTEW team could follow the samples in the future and until level 5 of Evaluation (Return on investment). That proposal was introduced during the End-of-Project Conference in Alicante, 2nd to 4th of September 2015 and all the ISTEW partner teams agreed. During WP10 lecture in Alicante, the Spanish team exposed other ideas to be discussed further: to also design a questionnaire for patients or health system users at levels 4 and 5 that could give a feedback of the improvements implemented and how they perceived those improvements, what was considered as „perceived improvements". That initiative would be useful to describe the social impact of HIS education in society and within communities (as it is stated by the Bled HIS definition

developed within ISTEW Project). The idea of developing a patient-centred evaluation framework was supported by partners.

Additionally, the Spanish Team suggested to include another part to the ISTEW Evaluation Framework separating between: measurable outcomes coming from HIS Education and the achieved impacts.

Another topic treated during the Alicante conference was to clearly design an Evaluation tool that could be used both ways: since the student starts HIS education (from what it is considered as the beginning of learning), through the whole ongoing til the return of investment (level 5) and also from a retrospective perspective, that's to say, a questionnaire appropriate to be completed from level 5 back to the beginning.

HIS Learning Evaluation Framework

Level 1. Reaction. STUDENT

Reaction evaluation is how the participants felt, and their personal reactions to the course.

1. Did you like the objectives planned for this course?
Please tick the one that most reflects your reaction

- ○ Very much
- ○ Somewhat
- ○ Undecided
- ○ Not Really
- ○ Not at all

Explain why:

1.1Would you add other objectives/contents to the course?

- ○ Yes
- ○ No

Please specify:

REFERENCES

- Parry, G. J., Carson-Stevens, A., Luff, D. F., McPherson, M. E., & Goldmann, D. A. (2013). Recommendations for evaluation of health care improvement initiatives. *Academic pediatrics*, *13*(6), S23-S30.
